# Preliminary experience with the transoral endoscopic thyroidectomy vestibular approach in selected complex cases: a case series and literature review

**DOI:** 10.3389/fsurg.2026.1882842

**Published:** 2026-07-27

**Authors:** Zeyu Li, Hui Li, Shiwei Zhou, Peng Wu, Yulong Tang, Xiaohua Song, Xiaowei Peng, Wu Li

**Affiliations:** 1 Department of Thyroid Surgery, The Affiliated Cancer Hospital of Xiangya School of Medicine, Central South University/Hunan Cancer Hospital, Changsha, Hunan, China; 2 Department of Thyroid Surgery, The Second Xiangya Hospital, Central South University, Changsha, Hunan, China

**Keywords:** case series, indications, literature review, neck masses, papillary thyroid carcinoma, thyroid nodules, thyroid surgery, transoral endoscopic thyroidectomy vestibular approach

## Abstract

**Background:**

The transoral endoscopic thyroidectomy vestibular approach (TOETVA) offers a scarless alternative to conventional thyroidectomy. Although established selection criteria exist, clinical scenarios exceeding these parameters are increasingly encountered in practice, yet evidence regarding their safety and feasibility remains limited. This study aimed to systematically review published indications for TOETVA and to preliminarily explore its feasibility in patients with conditions beyond conventional criteria through a case series analysis.

**Methods:**

A comprehensive literature review was conducted across PubMed, Web of Science, and the Cochrane Library for studies published up to July 2026, yielding 800 records, of which 131 studies met the inclusion criteria and were analyzed. Articles reporting specific inclusion criteria for TOETVA were analyzed, including thyroid size, gland volume, and maximum resected benign or malignant nodule sizes. In addition, a retrospective case series was performed involving seven patients who underwent TOETVA at a tertiary center between January 2021 and February 2024. Indications included bulky thyroid glands, large benign nodules, intrathyroidal T3a papillary thyroid carcinoma (PTC), and PTC with concomitant neck masses. Surgical feasibility, perioperative outcomes, complications, and recurrence were evaluated.

**Results:**

A total of 131 studies met the inclusion criteria. Most reports limited thyroid diameter to ≤10 cm and gland volume to ≤45–50 mL. For benign nodules, the most frequent upper limit was ≤6 cm, while for malignant tumors, ≤2 cm was the most common criterion. The case series included one bulky thyroid gland, one large benign nodule, two intrathyroidal T3a PTCs, and three PTCs with concomitant neck masses. All operations were successfully completed via TOETVA without conversion to open surgery. The mean operative time was 196 ± 25.3 min, mean drainage volume was 104 ± 67 mL, and mean postoperative hospital stay was 3.14 ± 1.68 days. One patient experienced transient hypoparathyroidism, and no recurrences or major complications were observed during a median follow-up of 34 months (range, 25–58 months).

**Conclusions:**

TOETVA is feasible for carefully selected patients beyond standard indications strictly within highly experienced centers, following thorough preoperative imaging and multidisciplinary team (MDT) evaluation. However, we strongly caution against a generalized expansion of indications. In these complex cases, oncologic safety must always be prioritized over cosmetic benefits, and further multicenter evidence is required to confirm long-term outcomes.

## 1 Introduction

With the increasing emphasis on aesthetic outcomes and the growing demand for minimally invasive procedures, endoscopic thyroidectomy has gained considerable attention in recent years (1, 2). Various techniques for endoscopic thyroidectomy have been developed, including approaches through the breast, axilla, subclavian area, and transoral vestibular route (3–10). Among these, the transoral endoscopic thyroidectomy vestibular approach (TOETVA) stands out as the only technique aligned with the principles of Natural Orifice Transluminal Endoscopic Surgery. TOETVA offers distinct advantages, such as a completely scarless exterior, minimal flap dissection, and comprehensive central lymph node clearance (11–16).

Since Anuwong et al.'s seminal report in 2015, which outlined the initial indications and contraindications for TOETVA (17), the technique has seen increasing global adoption. However, challenges such as limited visual field, complex operational angles, and prolonged operation times have hindered the widespread use and application of this technique (18, 19). In response, many surgeons have sought to optimize the original procedure to address these issues (20–23). These efforts aim to reduce surgical difficulty and expand the applicability of TOETVA, with various improvements contributing to enhanced safety and efficiency.

However, due to variations in surgical experience and skill levels among different surgeons, significant variability exists among research centers regarding the criteria for TOETVA. This variability is particularly pronounced concerning acceptable thyroid size, volume, and tumor dimensions for both benign and malignant lesions. Although the 2025 international consensus statement on remote-access thyroid surgery has established selection criteria for TOETVA (24), it also recognizes that patient eligibility may vary depending on surgeon skill, experience, and the specific technique employed. Consequently, clinical scenarios that fall outside strict parametric criteria are increasingly encountered in practice, yet evidence regarding their safety and feasibility remains limited. To address these issues, our study provides a structured narrative review of current TOETVA indications, integrating the latest findings from the literature. In addition, we present our clinical experience with seven unique cases that extend beyond conventional indications, including bulky thyroid gland, larger benign nodule, advanced malignancies, and concomitant neck masses. These cases explore the technical feasibility and oncologic safety of TOETVA in more complex scenarios. Through this study, we seek to provide valuable clinical insights that emphasize the necessity of meticulous patient selection and specialized surgical expertise, rather than advocating for a generalized expansion of TOETVA indications.

## 2 Materials and methods

### 2.1 Literature review

A comprehensive literature review was performed to identify relevant studies on the TOETVA published up to July 2026. Searches were conducted across PubMed, Web of Science, and the Cochrane Library databases. The search strategy combined both Medical Subject Headings and free-text keywords related to endoscopic thyroid surgery. The main terms included: “endoscopy,” “transoral endoscopic,” “vestibular,” “thyroidectomy,” and “thyroid surgery.” The complete search formula is provided in Supplementary Appendix 1.

### 2.2 Literature screening and data collection

All retrieved records from the Cochrane Library, Web of Science, and PubMed were imported into EndNote for reference management. Duplicate records were identified and removed. Literature screening and data extraction were performed independently by two researchers. Disagreements regarding study eligibility or data accuracy were resolved through discussion or, if necessary, by consulting a third senior reviewer.

The remaining articles were screened by title and abstract to exclude studies involving cadaveric or animal experiments, reviews or meta-analyses, letters or commentaries, case reports, non-English publications, and studies unrelated to TOETVA or thyroid diseases. Full texts of the potentially eligible studies were then reviewed to assess whether “explicit inclusion criteria” were provided. For the purposes of this study, “explicit inclusion criteria” were defined as clearly stated, reproducible parameters used by the original authors to select candidates for TOETVA, such as specific numerical thresholds for nodule diameter (cm), gland volume (mL), or specific pathological types.

Data extracted from the included studies encompassed the detailed inclusion criteria described in the methods, the maximum size of resected benign and malignant lesions reported in the results, the country and institution where the study was conducted, and the patient enrollment period. For studies reporting a range of enrollment years (e.g., 2017–2020), the final year of the range was used for classification.

### 2.3 Study cohort

This study retrospectively analyzed seven TOETVA cases treated at Hunan Cancer Hospital between January 2021 and February 2024, with a minimum follow-up duration of 25 months. All procedures were performed by a senior thyroid specialist and their dedicated surgical team. Prior to initiating these complex cases, the primary surgeon had long surpassed the learning curve, with a cumulative experience of over 1,000 TOETVA surgeries and an annual volume of approximately 300 cases. These seven cases were selected because they represent a diverse range of clinical scenarios that extend beyond standard TOETVA indications. The cases were chosen specifically due to their complexity, including factors such as bulky thyroid glands, larger benign nodules, intrathyroidal T3a papillary thyroid carcinoma (PTC), and concomitant neck masses, all of which posed unique surgical challenges. By selecting these cases, we aimed to explore the feasibility and safety of TOETVA in situations where it is typically considered less suitable. These cases also offered valuable insights into the adaptability of the technique when addressing more complex thyroid pathologies. This study was conducted in accordance with the Declaration of Helsinki. Ethical approval was obtained from the Medical Ethics Committee of Hunan Cancer Hospital, and written informed consent was secured from all participants. This case series is reported in accordance with the STROBE Guidelines (25).

### 2.4 Case identification and perioperative management

All cases were identified through the hospital's electronic medical record system using surgical codes and corresponding pathology reports to ensure case accuracy. As the transoral route is classified as a clean-contaminated (Class II) incision, routine preoperative oral cavity preparation was performed. A single dose of intravenous Cefazolin (2 g) was administered 30 min before the skin incision as antibiotic prophylaxis, followed by one additional postoperative dose. Postoperatively, patients were instructed to rinse with chlorhexidine mouthwash to maintain oral hygiene.

### 2.5 Data collection and follow-up

Data collected included patient demographics (age, gender, BMI), maximum nodule diameter, operative time, intraoperative blood loss, postoperative pathology results, number of lymph nodes retrieved and those found positive, postoperative drainage volume, length of hospital stay, postoperative complications, and recurrence rates. All patients underwent preoperative and postoperative laryngoscopy to evaluate vocal cord function and document recurrent laryngeal nerve integrity. Mental nerve function was assessed by clinical examination of chin and lower lip sensation at postoperative day 1, at discharge, and during each follow-up visit. Surgical site infection and oral vestibular complications were monitored through daily inspection of the oral vestibule and cervical region during hospitalization, as well as at each outpatient follow-up. Postoperative survival and recurrence data were obtained through outpatient medical records and telephone follow-up. Recurrence was defined as the detection of biopsy-proven locoregional or distant disease, or the appearance of new suspicious lesions on imaging that were confirmed by cytology or demonstrated progressive growth during follow-up. Patients were followed at 3, 6, and 12 months postoperatively, and annually thereafter. Each follow-up included medical history review, physical examination, routine blood tests, and neck ultrasound or CT.

### 2.6 Preoperative assessment and selection criteria

Preoperative evaluation involved detailed neck ultrasound and contrast-enhanced CT to meticulously assess tumor characteristics, including capsular or posterior involvement, recurrent laryngeal nerve risk, and both central and lateral neck nodal status. Furthermore, systemic and anatomical factors—such as BMI, severe Hashimoto's thyroiditis, and oral vestibular anatomy—were systematically evaluated to ensure technical feasibility. Finally, surgical candidacy for all expanded indications was strictly established through comprehensive multidisciplinary team (MDT) discussions.

Based on this rigorous assessment, the specific inclusion criteria comprised: (1) benign thyroid nodules exceeding the conventional TOETVA indication size of 6 cm, or a total thyroid gland dimension exceeding 10 cm or volume exceeding 45 mL; (2) PTC classified as T3a, all of which underwent thorough MDT evaluation confirming intrathyroidal confinement without evidence of capsular invasion or preoperative clinical nodal metastasis (cN0); and (3) PTC accompanied by other neck masses amenable to simultaneous resection. In all PTC cases, prophylactic central neck dissection was performed. Exclusion criteria included a history of previous neck surgery or incomplete medical records.

### 2.7 Surgical procedure and specimen extraction in TOETVA

All procedures were performed under general anesthesia with endotracheal intubation. The patient was placed in the supine position with neck extension. The transoral vestibular incision was designed according to our previously described technique. Briefly, a midline mucosal incision was extended horizontally to expose and identify the bilateral mental nerve branches before port placement, allowing their preservation during subsequent dissection and facilitating specimen extraction (26, 27). After creation of the subplatysmal working space with CO₂ insufflation (6–8 mmHg), three vestibular ports were inserted. A 10 mm central port was placed at the midline as the observation port, and two 5 mm lateral working ports were positioned approximately 2.5 cm from the central port, with their locations adjusted according to the course of the mental nerves. Thyroidectomy and indicated central lymph node dissection were performed under endoscopic visualization. Intraoperative neuromonitoring was routinely applied in total thyroidectomy and in patients with complex anatomy or extended procedures, whereas it was not routinely used for conventional unilateral lobectomy. The recurrent laryngeal nerve was identified and its functional integrity was confirmed when Intraoperative neuromonitoring was applied. The parathyroid glands were carefully identified and preserved with their vascular supply whenever possible. Autotransplantation was performed when parathyroid viability was considered compromised. All specimens were placed in a retrieval bag before extraction. For malignant tumors, the specimen was removed en bloc without fragmentation to maintain oncological integrity. For benign lesions, specimen fragmentation within the retrieval bag was permitted when necessary to facilitate removal. In cases requiring extraction of bulky specimens, the vestibular/submental extraction pathway was selectively enlarged after careful identification and protection of the mental nerves, according to our previously reported technique (26, 27). Conversion to open surgery was considered when complete oncologic resection could not be achieved, uncontrolled bleeding occurred, critical anatomical structures could not be safely identified or preserved, or continuation of the endoscopic procedure was considered unsafe.

### 2.8 Statistical analysis

Data were analyzed using SPSS (version 23.0; IBM Corp., Armonk, NY, USA). Continuous variables were presented as mean ± standard deviation, and categorical variables as counts and percentages. Given the limited sample size, only descriptive statistics were applied. Data from the literature review were summarized as frequencies and proportions. Graphical visualization was performed using GraphPad Prism (version 8.3).

## 3 Results

### 3.1 Literature selection process

**Figure 1:**
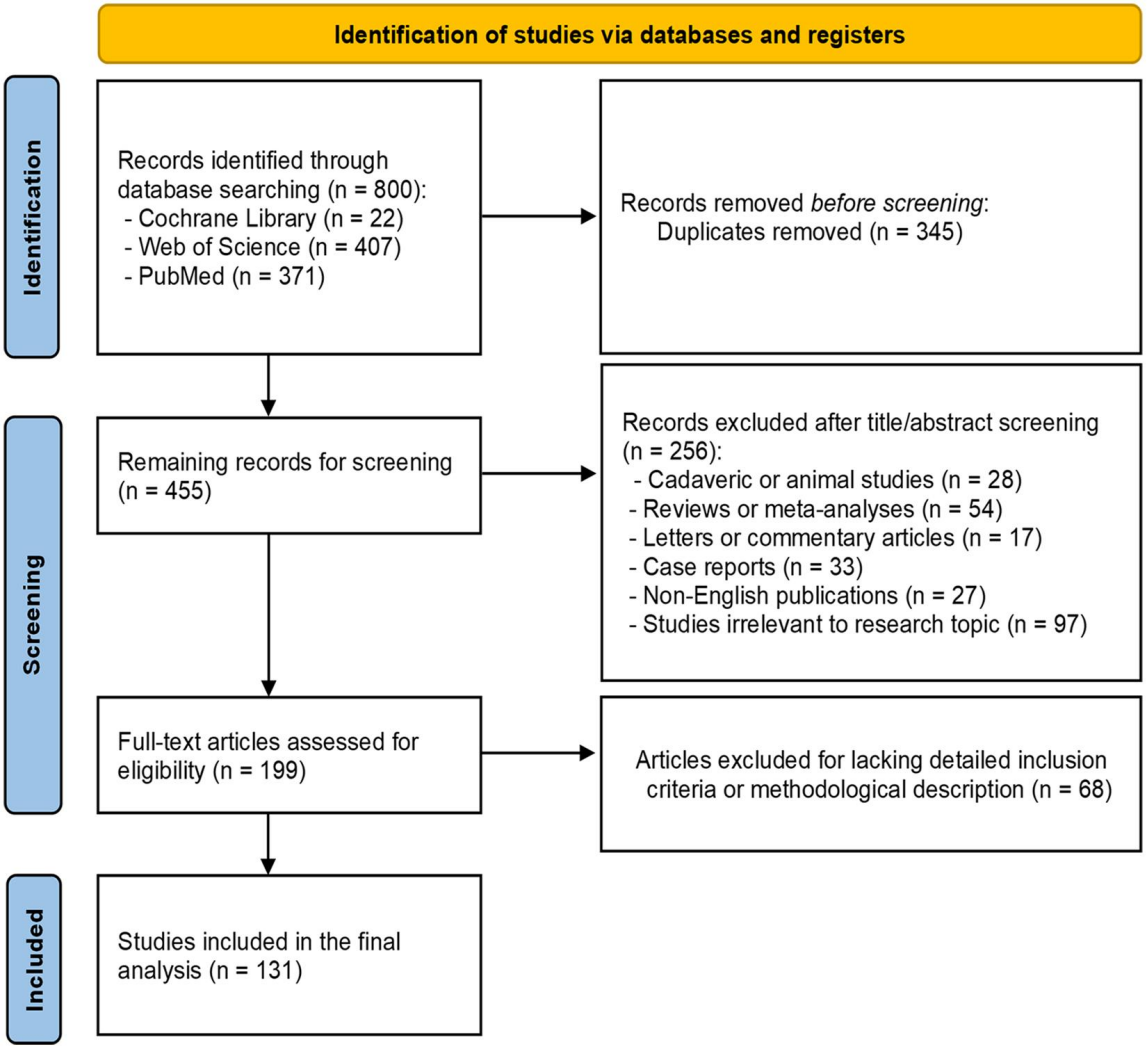
Flow diagram shows the process of study selection.

A total of 800 records were identified from the Cochrane Library, Web of Science, and PubMed. After removing 345 duplicates, 455 unique articles remained for screening. Based on titles and abstracts, 256 studies were excluded, including cadaveric or animal studies, reviews or meta-analyses, letters or commentaries, case reports, non-English publications, and studies unrelated to the research topic. The remaining 199 full-text articles were assessed for eligibility, and 68 were excluded due to insufficient inclusion criteria or methodological description. Finally, 131 studies were included in the final analysis (Figure 1).

### 3.2 Literature analysis

A total of 131 studies explicitly reported inclusion criteria for TOETVA. Among these, 26 articles provided quantitative data on thyroid gland volume and 25 on maximum diameter, while 86 and 108 studies described inclusion thresholds for benign and malignant nodules, respectively. In addition, 48 studies reported the maximum size of resected nodules, including both benign and malignant lesions (Supplementary Table S1).

**Figure 2:**
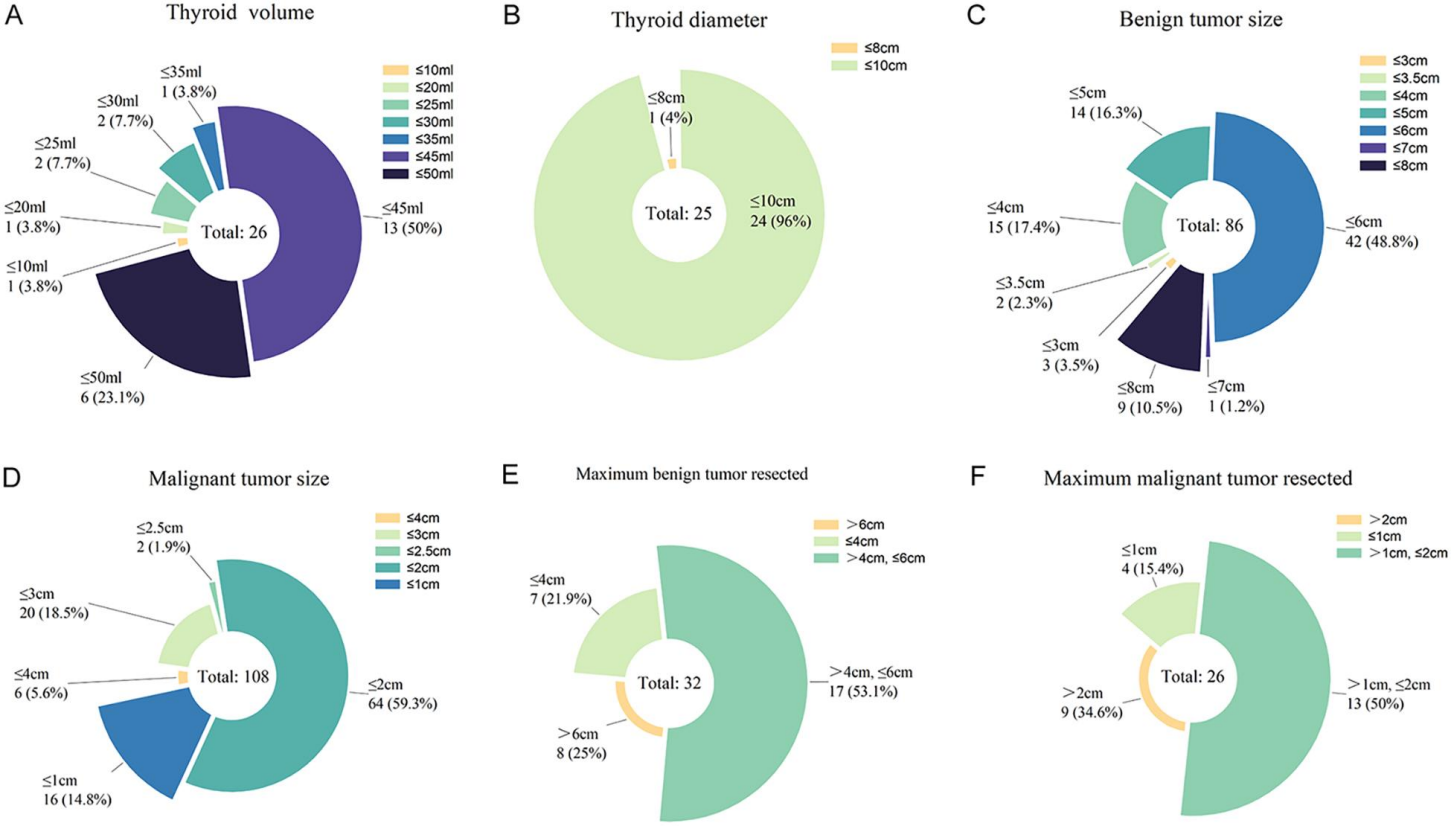
In the literature included in the literature review, the total number and distribution range of articles reporting the inclusion criteria of TOETVA for thyroid size, volume, and the size of benign and malignant tumors, as well as the maximum sizes of benign and malignant tumors removed through TOETVA surgery. **(A)** The total number and distribution range of studies reporting the limitations imposed by TOETVA on thyroid volume. **(B)** The total number and distribution range of studies reporting the limitations imposed by TOETVA on thyroid diameter. **(C)** The total number and distribution range of studies reporting the limitations imposed by TOETVA on the size of benign tumors. **(D)** The total number and distribution range of studies reporting the limitations imposed by TOETVA on the size of malignant tumors. **(E)** The total number and distribution range of studies reporting the maximum size of benign tumors removed through TOETVA. **(F)** The total number and distribution range of studies reporting the maximum size of malignant tumors removed through TOETVA.

Regarding thyroid gland parameters, one study each reported a volume threshold of ≤10 mL and ≤20 mL, two studies reported ≤25 mL, another two reported ≤30 mL, one reported ≤35 mL, thirteen reported ≤45 mL, and six reported ≤50 mL (Figure 2A). In terms of diameter, 24 studies set the upper limit at ≤10 cm and one study reported ≤8 cm (Figure 2B).

For benign thyroid nodules, 86 studies defined explicit inclusion criteria. Three studies limited benign nodules to ≤3 cm, two to ≤3.5 cm, fifteen to ≤4 cm, fourteen to ≤5 cm, forty-two to ≤6 cm, one to ≤7 cm, and nine to ≤8 cm (Figure 2C). Overall, the majority of studies (approximately 90%) adopted 6 cm or smaller as the upper threshold for benign nodules.

Regarding malignant tumors, 108 studies reported tumor size–based criteria. Sixteen studies restricted tumor size to ≤1 cm, sixty-four to ≤2 cm, two to ≤2.5 cm, twenty to ≤3 cm, and six to ≤4 cm (Figure 2D).

In addition, 48 studies provided data on the maximum size of resected nodules. Among 32 studies reporting resected benign nodules, eight described nodules larger than 6 cm, while twenty-four reported nodules ≤6 cm, including seventeen with nodules >4 cm and ≤6 cm, and seven with nodules ≤4 cm (Figure 2E). For malignant lesions, 26 studies documented the largest resected tumors: nine reported tumor sizes >2 cm, while seventeen reported tumors ≤2 cm, including thirteen studies with tumors between >1 and ≤2 cm, and four studies with tumors ≤1 cm (Figure 2F).

### 3.3 Reported inclusion criteria across countries

A total of 131 studies were included in our review; among them, data from the seven countries or regions with the highest publication volumes were specifically extracted to summarize the primary reported inclusion criteria for TOETVA (Supplementary Table S2). China reported the largest number of publications (*n* = 48), with malignant tumor limits generally set at ≤1–4 cm, benign nodules at ≤3.5–6 cm, thyroid diameters ≤8–10 cm, and gland volumes ≤25–50 mL. Korea (*n* = 18) reported malignant tumor limits of ≤2–4 cm and benign nodules of ≤4–8 cm. Vietnam (*n* = 14) adopted malignant tumor limits of ≤2 cm, benign nodules ≤6 cm, thyroid diameters ≤10 cm, and gland volumes ≤10–45 mL. India (*n* = 10) reported malignant tumor limits of ≤2–4 cm, benign nodules ≤3–6 cm, and thyroid volumes ≤30–45 mL. The United States (*n* = 7) described malignant tumors ≤2–3 cm, benign nodules ≤6 cm, thyroid diameters ≤10 cm, and volumes ≤45 mL. Taiwan (*n* = 7) reported malignant tumors ≤1–2 cm and benign nodules ≤5–8 cm. Turkey (*n* = 6) reported malignant tumors ≤1–3 cm, benign nodules ≤4–6 cm, thyroid diameters ≤10 cm, and volumes ≤45–50 mL.

### 3.4 Summary of the case serials

**Figure 3:**
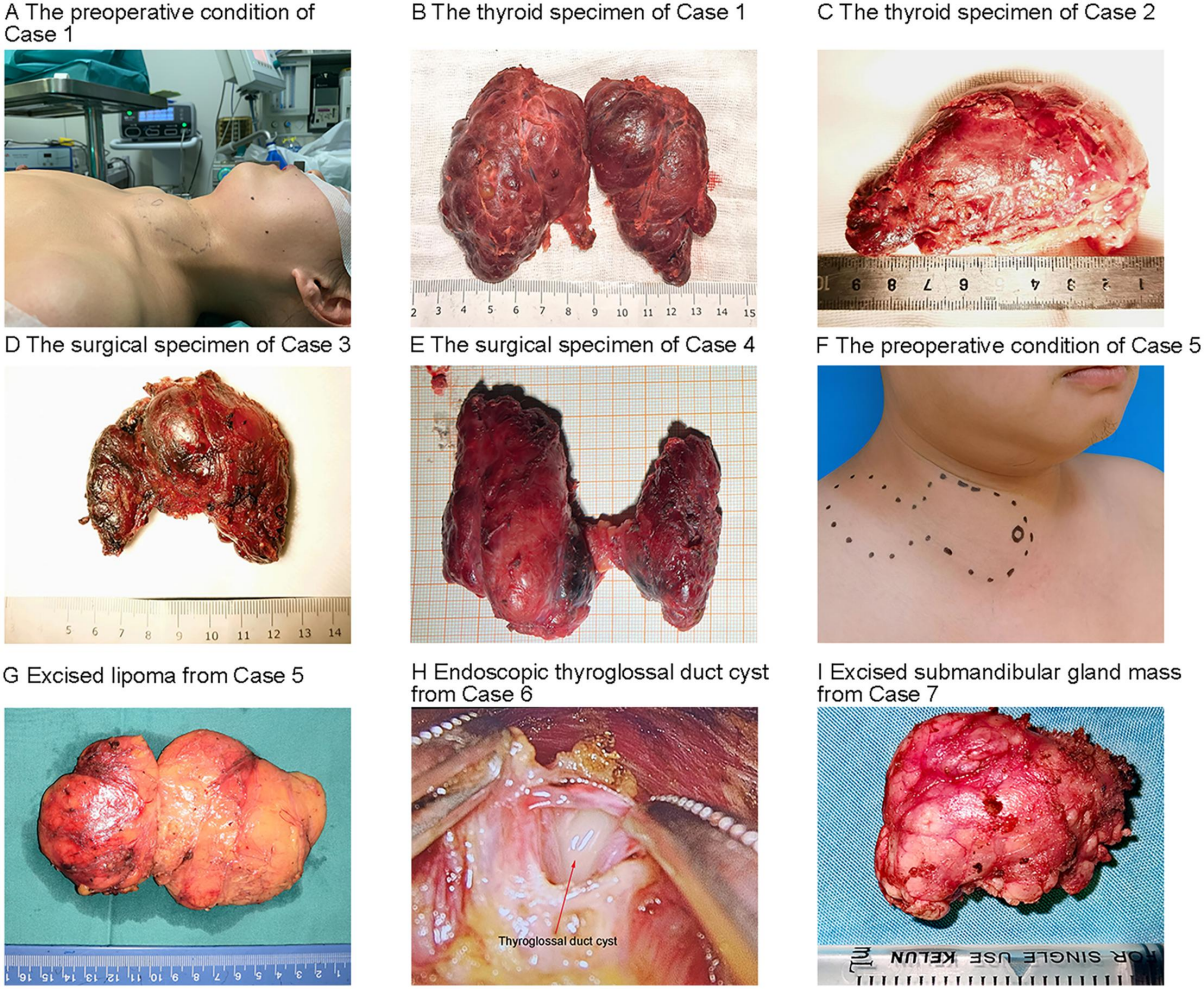
**(A)** the preoperative condition of case 1. The thyroid specimen of Case 1 **(B)** and Case 2 **(C)**. The surgical specimen of Case 3 **(D)** and Case 4 **(E)**. **(F)** The preoperative condition of Case 5. **(G)** Excised lipoma from Case 5. **(H)** Endoscopic thyroglossal duct cyst from Case 6 indicated by the red arrow. **(I)** Excised submandibular gland mass from Case 7.

Our study included seven cases with diverse thyroid pathologies. Case 1 involved a bulky thyroid gland, while Case 2 presented with a large benign thyroid nodule measuring 7.6 cm (Figures 3A–C). Cases 3 and 4 had intrathyroidal T3a PTC (4.9 cm and 5.6 cm, respectively), with MDT-confirmed absence of gross extrathyroidal extension and no imaging evidence of nodal metastasis/matting (Figures 3D,E).

Additionally, three cases featured PTC accompanied by other masses: Case 5 involved a 10 cm lipoma (Figures 3F,G), Case 6 had a thyroglossal duct cyst (Figure 3H), and Case 7 presented with a 3 cm submandibular gland mass (Figure 3I).

The cohort comprised one male and six female patients, with a mean age of 35.7 ± 12.6 years and a mean BMI of 23.9 ± 2.87 kg/m². The average maximum nodule size across all cases was 37.1 ± 27.2 mm (Table 1).

**Table 1:** Demographics data of the patients (*n* = 7).

Number	Sex (M/F)	Age (years)	BMI	Tumor size (mm)	Thyroid volumes (mL)	Preoperative diagnosis	Special case comments
1	F	32	20.83	50	71	Nodular goiter and diffuse thyroid enlargement	Thyroid sizes and volumes > 10 cm, 45 mL
2	F	54	20.96	76	85	Follicular tumor of the right lobe	Benign nodule with a maximum diameter >7 cm
3	F	20	25.91	49	38	Bilateral PTC	Malignant nodule with a maximum diameter >4 cm
4	F	25	21.30	56	42	Bilateral PTC	Malignant nodule with a maximum diameter >5 cm
5	M	29	27.68	12	15	Right PTC combined with a right neck mass	TOETVA combined with central neck dissection and lipoma excision
6	F	49	26.37	8	12	Left PTC combined with a thyroglossal duct cyst	TOETVA with central neck dissection and thyroglossal duct cyst excision
7	F	41	24.44	9	15	Bilateral PTC combined with right submandibular gland mass	TOETVA with central neck dissection and submandibular gland mass excision

TOETVA, transoral endoscopic thyroidectomy vestibular approach; PTC, papillary thyroid carcinoma; BMI, body mass index.

### 3.5 Perioperative and postoperative outcomes

All surgeries were completed via TOETVA without conversion to open surgery. The mean operation time was 196 ± 25.3 min, with a mean blood loss of 21.4 ± 10.7 mL. Regarding oncologic efficacy, final pathology confirmed negative surgical margins (R0 resection) and intact tumor capsules in all five malignant cases (Cases 3–7). Regarding lymph node status, a clear distinction was observed between preoperative clinical evaluation and postoperative pathological findings. Cases 1 and 2 involved benign nodules and did not undergo lymph node dissection. All five patients with malignant disease (Cases 3–7) were evaluated as clinically node-negative (cN0) preoperatively and subsequently underwent central neck dissection. Postoperative pathology revealed occult central lymph node metastasis in two patients (Cases 3 and 4), reclassifying their nodal status to pN1a. The specific ratios of positive to retrieved lymph nodes for these two cases were 13/27 and 17/21, respectively. The remaining three malignant cases (Cases 5, 6, and 7) were confirmed as pathologically node-negative (pN0), with 0/6, 0/10, and 0/23 positive nodes, respectively. The average drainage volume during the first three postoperative days was 104 ± 67 mL, and the mean postoperative hospital stay was 3.14 ± 1.68 days. Case 1 experienced transient hypoparathyroidism postoperatively, which resolved within one month. No vestibular wound infections or other significant complications were noted in any of the cases. All patients underwent preoperative and postoperative laryngoscopy to assess vocal cord function; no recurrent laryngeal nerve injuries were documented. Mental nerve function was assessed by clinical examination of chin and lower lip sensation; no persistent hypoesthesia was observed. Quality-of-life measures were not formally administered in this retrospective series; however, all patients reported satisfaction with cosmetic outcomes at final follow-up. All patients strictly adhered to postoperative neck rehabilitation exercises, and two patients (Cases 3 and 4) underwent adjuvant I¹³¹ therapy. The median follow-up period was 34 months (range, 25–58 months), with no observed recurrences (Table 2).

**Table 2:** Perioperative data of the patients (*n* = 7).

Number	Operation time (min)	Blood loss (mL)	Resected thyroid volume (mL)	Pathological diagnosis	Positive and retrieved lymph nodes (VI region)	Multifocality	ATA risk stratification	Drainage volume (the first three days, mL)	Complications	Postoperative hospitalization (day)	Follow-up duration (month)
1	180	20	71	Nodular goiter	–	No	NA	70	Transient hypoparathyroidism	2	32
2	160	20	77	Follicular thyroid adenoma	–	No	NA	130	–	2	33
3	218	30	38	PTC	13/27	Yes	Intermediate-High^a^	85	–	3	34
4	235	40	42	PTC and HT	17/21	Yes	Intermediate-High^a^	230	–	6	25
5	180	10	8	PTC and lipoma	0/6	No	Low	80	–	5	52
6	200	10	6	PTC and thyroglossal duct cyst	0/10	No	Low	15	–	2	58
7	200	20	15	PTC and submandibular gland mass	0/23	No	Low	120	–	2	37

PTC, papillary thyroid carcinoma; HT, Hashimoto's thyroiditis.

a Indicates patients who subsequently received postoperative adjuvant radioactive iodine (I¹³¹) therapy.

## 4 Discussion

Since the introduction of TOETVA, early studies have predominantly focused on its application in small to medium-sized thyroid nodules and low-risk PTC patients (11, 28–36). As a novel minimally invasive surgical technique, TOETVA initially adopted strict selection criteria based on factors such as nodule size, thyroid volume, and tumor staging (37). These stringent criteria reflect a high level of concern for patient safety and surgical outcomes, aiming to ensure optimal results and minimize complication risks.

Most studies restrict benign nodules to a maximum diameter of approximately 6 cm, set malignant tumor thresholds at ≤2 cm, and limit thyroid volume to ≤45 mL (38–41). However, the inclusion criteria reported worldwide exhibit variability among countries and regions (Supplementary Table S2). China has contributed the largest number of studies, with malignant tumor limits generally set at ≤1–4 cm and benign nodules at ≤3.5–6 cm, while Korea, India, and Turkey reported similar or slightly expanded ranges in selected cases. In contrast, Vietnam, the United States, and Taiwan maintained more conservative thresholds, typically restricting malignant tumors to ≤2–3 cm. These differing thresholds likely reflect local institutional experience, specific surgeon expertise, and inherent reporting bias, rather than universally proven oncologic or surgical safety limits. With continuous advancements in endoscopic technology and accumulating surgical experience, some centers have cautiously explored expanding TOETVA indications, particularly for larger benign thyroid nodules. Several reports have demonstrated the technical feasibility of performing TOETVA in such cases (1, 17, 19, 42–46). For example, Park and Sun (19) in 2017 successfully removed benign nodules up to 7.5 cm in diameter using this approach; Anuwong et al. (1) in 2018 reported successful TOETVA for benign nodules as large as 10 cm; and Bamroong et al. (47) in 2020 described cases of benign nodules up to 13 cm removed using modified instruments to facilitate the procedure. These encouraging experiences suggest that TOETVA may be technically feasible in selected patients with large benign thyroid nodules. Nonetheless, they serve as preliminary references for clinical decision-making when such scenarios arise, requiring rigorous individualized evaluation.

The main reason for limiting the size of benign nodules is that the central incision for TOETVA is typically small, making it challenging to effectively remove larger nodules through this incision (1, 19). Attempting to remove larger nodules increases surgical difficulty and may lead to complications such as damage to the mental nerve (48). Some researchers have addressed this issue by adding additional surgical access points (such as submental, retroauricular, or axillary approaches) (49–51). Although these methods expand the indications and enable the removal of larger specimens, the extra incisions increase surgical trauma and leave scars on the skin, which may be unsuitable for patients with high cosmetic demands.

To address the challenge of removing larger nodules, some literature suggests strategies such as expanding the central incision to facilitate specimen extraction (52). In extreme cases, there have been reports of segmenting specimens into smaller pieces and placing them in specimen bags for extraction (1, 17, 19, 47, 53). However, it is important to note that enlarging the incision for specimen removal may increase the risk of mental nerve damage, which limits the applicability of this approach (48).

In this context, our center proposed a single-incision design that exposes the mental nerve to better remove larger specimens while reducing the risk of mental nerve damage (26). This innovative approach allows for the extension of the surgical tunnel during specimen extraction, enabling larger thyroid nodules to be safely removed through the central incision. In our series of cases, this design was successfully applied to patients with large benign nodules, and no mental nerve damage or other complications were observed. A mild transient discomfort—including temporary chin stiffness or numbness—was noted in some patients, likely due to mechanical traction on the mentalis muscle and surrounding tissues during specimen retrieval. However, these symptoms completely resolved within three months postoperatively. Future refinements of this technique and appropriate postoperative rehabilitation may further enhance patient comfort and recovery.

When addressing malignant tumors, the use of TOETVA presents distinct challenges, particularly in managing larger tumors where considerations of size and invasiveness become critical (48). Successfully treating malignant tumors requires not only the complete removal of malignant tissues but also minimizing the impact on adjacent normal structures (54–56). Current guidelines for TOETVA emphasize strict inclusion criteria, typically recommending its use for malignant thyroid tumors smaller than 4 cm, corresponding to the T2 stage in the TNM staging system. For added safety, many studies advocate an even more restrictive criterion of ≤2 cm, aligning with the T1 stage (57).

Despite these challenges, there are encouraging reports of successful TOETVA procedures in selected patients with PTC larger than 4 cm in diameter. Wang et al. (58) in 2018 reported cases involving malignant nodules up to 4.0 cm in size, and Zheng et al. (35) in 2021 documented PTC cases with tumor diameters reaching 4.3 cm. Although such cases remain uncommon, they suggest that TOETVA may be feasible for tumors exceeding conventional size limits under carefully evaluated conditions. In our series, two patients with intrathyroidal T3a PTC (4.9 cm and 5.6 cm) chose TOETVA for cosmetic considerations, and the procedures were performed after comprehensive preoperative imaging assessments and MDT discussions, which evaluated critical factors such as tumor location, capsule integrity, and potential surrounding invasion. Both surgeries were successfully completed without postoperative complications, and no recurrence was observed during follow-up.

In the present series, among patients with preoperative cN0 T3a PTC who underwent prophylactic central neck dissection, postoperative pathology revealed central lymph node metastasis in a substantial proportion of cases. This finding is consistent with the well-recognized phenomenon of occult nodal metastasis in PTC, which may not be reliably detected by preoperative imaging. Notably, larger primary tumor size has been associated with an increased risk of occult central lymph node metastasis, even in patients with clinically negative necks (59). These observations suggest that for patients with cT3a PTC and clinically negative nodes, the risk of occult central metastasis is non-negligible. Consequently, when TOETVA is considered for such cases, particular emphasis should be placed on ensuring the completeness of central neck dissection to achieve adequate oncological clearance. Furthermore, coexisting Hashimoto's thyroiditis, as in Case 4, warrants special consideration. Chronic autoimmune inflammation in Hashimoto's thyroiditis often causes reactive hyperplasia of central compartment lymph nodes, which increases the harvestable nodal yield—largely explaining the high number of nodes retrieved (21 nodes) in this case. Additionally, Hashimoto's thyroiditis impacts postoperative surveillance; elevated anti-thyroglobulin antibodies can interfere with serum thyroglobulin assays. Thus, monitoring for recurrence cannot rely solely on thyroglobulin levels, but must incorporate dynamic anti-thyroglobulin antibodies tracking and serial neck ultrasonography.

In addition to its application for malignant thyroid tumors, TOETVA's potential for managing concurrent neck masses is an area of growing interest, despite limited literature on the topic. Our study provides valuable insights into this application, demonstrating the effectiveness of TOETVA in simultaneously addressing PTC and other neck masses within the same procedure. This advantage can be attributed to the unique superior-to-inferior view offered by the transoral vestibular approach, which allows excellent visualization of the central neck and facilitates access to adjacent or concomitant lesions without additional incisions (60). This approach underscores TOETVA's versatility and its capability to manage both thyroid conditions and additional neck masses in a single, minimally invasive operation.

For patients with PTC combined with other neck masses, TOETVA not only offers aesthetic benefits but also consolidates the treatment of multiple conditions into a single procedure. This approach reduces the number of surgeries required and alleviates the physical and psychological stress associated with repeated interventions. Postoperative follow-ups in our cases showed no significant complications or tumor recurrence, confirming that TOETVA is both safe and feasible for this patient subset. This method aligns well with modern healthcare's emphasis on personalized and patient-centered care (61).

In addressing both intrathyroidal T3a tumors and concurrent neck masses with TOETVA, our study advocates for nuanced strategies and close multidisciplinary collaboration. The successful implementation of TOETVA in these complex, carefully selected cases highlights the critical role of an MDT approach, which facilitates collaborative discussions among surgeons, radiologists, pathologists, endocrinologists, and other specialists and allows for the development of personalized, comprehensive treatment plans (62, 63).

MDT discussions must go beyond tumor size and invasion depth to include a holistic assessment of the patient's overall condition and the feasibility of the proposed surgical approach. Thorough preoperative evaluation is essential to determine the optimal treatment strategy, such as the choice of surgical approach, the need for postoperative I¹³¹ therapy, and the anticipated risk of complications, while carefully weighing the aesthetic benefits of TOETVA against its oncologic and procedural risks. Within this framework, refining patient selection criteria and adopting a systematic, individualized selection process are crucial for optimizing outcomes, ensuring patient safety, and mitigating the risks associated with extending TOETVA beyond its conventional indications. In addition, applying the concept of whole-course management, with structured postoperative follow-up and imaging surveillance, helps maintain continuity of care and facilitates the timely detection of recurrence or treatment-related issues.

Thus, while these findings suggest that TOETVA may be feasible in carefully selected complex cases at experienced centers, we emphasize that such applications should not be interpreted as a generalized expansion of indications. Inadequate preoperative evaluation and insufficient surgical experience may increase procedural complexity, prolong operative time, raise the risk of intraoperative bleeding, and impair the management of unexpected intraoperative events, ultimately compromising surgical safety and postoperative recovery (64–66). Careful patient selection, meticulous preoperative assessment, and adequate surgical expertise are therefore essential prerequisites for safely extending TOETVA beyond standard indications. At the same time, although the cosmetic advantages of TOETVA are undeniably attractive to many patients, aesthetic benefits should never override oncologic safety. In thyroid cancer surgery, treatment decisions must remain grounded in oncologic principles, as prioritizing cosmetic outcomes at the expense of adequate tumor control may lead to suboptimal long-term results.

This study has several limitations. First, the relatively small sample size may restrict the generalizability of our findings, and larger-scale studies with more diverse patient populations are needed to validate these results. Second, the short follow-up duration limits the assessment of long-term outcomes, including recurrence rates and the durability of both aesthetic and oncologic benefits; longer follow-up would provide a more comprehensive understanding of TOETVA's long-term efficacy. In addition, endoscopic thyroid surgery techniques, including TOETVA, are rapidly evolving, and our findings are based on current technology and institutional experience, which may change over time. Finally, this study focuses primarily on clinical and surgical outcomes and does not include detailed patient-reported measures assessed with validated structured tools, such as quality of life and cosmetic satisfaction, which are essential for a holistic evaluation of surgical success.

## 5 Conclusions

In conclusion, this study provides a comprehensive overview of current TOETVA indications and explores its potential application in complex cases beyond conventional selection criteria. While our experience demonstrates that TOETVA is technically feasible in carefully selected patients, the assessment of oncologic safety remains preliminary given the small sample size and short follow-up duration. We strongly caution against a generalized expansion of its indications. Such complex procedures must be strictly limited to highly experienced centers where oncologic principles and functional preservation remain the highest priorities. Importantly, open thyroidectomy remains the most reproducible and standard approach for high-risk malignant disease. Further multicenter studies with longer follow-up are needed to confirm these findings and refine standardized patient selection protocols.

## Data Availability

The raw data supporting the conclusions of this article will be made available by the authors, without undue reservation.
