# Supplementary material for: Preliminary experience with the transoral endoscopic thyroidectomy vestibular approach in selected complex cases: a case series and literature review
Source: Front Surg. 2026 Jul 27;13:1882842. doi: 10.3389/fsurg.2026.1882842 (PMC13454321; doi:10.3389/fsurg.2026.1882842)
Supplement: Supplementary file 1 [file Supplementaryfile1.docx]

Search Strategies Used in the Literature Review

PubMed

| Step | Search Query | Description / Keywords |
| --- | --- | --- |
| #1 | ((((("Endoscopy"[Mesh]) OR (((((((Endoscopy[Title/Abstract]) OR ("Endoscopic Surgical Procedure"[Title/Abstract])) OR ("Endoscopic Surgical Procedures"[Title/Abstract])) OR ("Endoscopy, Surgical"[Title/Abstract])) OR ("Surgical Endoscopy"[Title/Abstract])) OR ("Surgical Procedure, Endoscopic"[Title/Abstract])) OR ("Surgical Procedures, Endoscopic"[Title/Abstract]))) AND (transoral[Title/Abstract])) AND (vestibular[Title/Abstract])) AND (("Thyroidectomy"[Mesh]) OR ((Thyroidectomy[Title/Abstract]) OR (Thyroidectom*[Title/Abstract])))) OR (TOETVA[Title/Abstract]) | Combined MeSH and free-text terms for TOETVA-related thyroidectomy via the transoral vestibular approach. |

Web of Science

| Step | Search Query | Description / Keywords |
| --- | --- | --- |
| #1 | TS = (Endoscopy OR "Endoscopic Surgical Procedure" OR "Endoscopic Surgical Procedures" OR "Endoscopy, Surgical" OR "Surgical Endoscopy" OR "Surgical Procedure, Endoscopic" OR "Surgical Procedures, Endoscopic") | Free-text terms related to endoscopic surgery |
| #2 | TS = (vestibular) | Add vestibular term |
| #3 | TS = (transoral) | Add transoral term |
| #4 | TS = (Thyroidectomy) | Free-text term for thyroidectomy |
| #5 | #1 AND #2 AND #3 AND #4 | Combine endoscopy, vestibular, transoral, and thyroidectomy terms |
| #6 | TS = (TOETVA) | Include acronym term (TOETVA) to capture studies using the abbreviated form |
| #7 | #5 OR #6 | Final combined search set for TOETVA |

Cochrane Library

| Step | Search Query | Description / Keywords |
| --- | --- | --- |
| #1 | MeSH descriptor: [Endoscopy] explode all trees | Controlled vocabulary term for endoscopy |
| #2 | MeSH descriptor: [Thyroidectomy] explode all trees | Controlled vocabulary term for thyroidectomy |
| #3 | (Endoscopy):ti,ab,kw OR ("Endoscopic Surgical Procedure"):ti,ab,kw OR ("Endoscopic Surgical Procedures"):ti,ab,kw OR ("Endoscopy, Surgical"):ti,ab,kw OR ("Surgical Endoscopy"):ti,ab,kw OR ("Surgical Procedure, Endoscopic"):ti,ab,kw OR ("Surgical Procedures, Endoscopic"):ti,ab,kw | Free-text terms related to endoscopic surgery |
| #4 | #1 OR #3 | Combine MeSH and free-text terms for endoscopy |
| #5 | ("Thyroidectomy"):ti,ab,kw OR (thyroidectom*):ti,ab,kw | Free-text terms for thyroidectomy |
| #6 | #2 OR #5 | Combine MeSH and free-text terms for thyroidectomy |
| #7 | (vestibular):ti,ab,kw | Add vestibular term |
| #8 | (transoral):ti,ab,kw | Add transoral term |
| #9 | #4 AND #6 AND #7 AND #8 | Combine endoscopy, thyroidectomy, vestibular, and transoral terms |
| #10 | (TOETVA):ti,ab,kw | Include acronym term (TOETVA) |
| #11 | #9 OR #10 | Final combined search set for TOETVA |
