# Supplementary material for: Preliminary experience with the transoral endoscopic thyroidectomy vestibular approach in selected complex cases: a case series and literature review
Source: Front Surg. 2026 Jul 27;13:1882842. doi: 10.3389/fsurg.2026.1882842 (PMC13454321; doi:10.3389/fsurg.2026.1882842)
Supplement: Supplementary file 2 [file Table1.docx]

**Supplementary Table 1**. Summary of Inclusion Criteria and Resected Lesion Characteristics of the Included Studies (n = 121)

| Publication Year | Inclusion Criteria | | | | Largest Resected Lesion | |
| --- | --- | --- | --- | --- | --- | --- |
|  | Benign Nodule (cm) | Malignant Tumor (cm) | Thyroid Volume (ml) | Maximum Thyroid Diameter (cm) | Benign (cm) | Malignant (cm) |
| 2015 | NA | NA | NA | NA | NA | NA |
| 2016 | NA | ^1,2^ | NA | ^1–3^ | ^2^ | ^1^ |
| 2017 | ^4–6^ | ^4,6^ | ^6^ | ^6^ | ^4–6^ | ^4,6^ |
| 2018 | ^7–14^ | ^7,9,10,14–16^ | ^8,11,12^ | ^11,16^ | ^7–10,16^ | ^8,10,15^ |
| 2019 | ^17–21^ | ^17–20,22^ | ^18^ | ^18^ | ^19–21^ | ^19,22^ |
| 2020 | ^23–38^ | ^23,24,26–29,31–43^ | ^27,28,34^ | ^24,27,28,31,34,43^ | ^27,29,31,34,38,43^ | ^36,39^ |
| 2021 | ^44–59^ | ^44,45,47,49,51,53–63^ | ^44,51,58,59^ | ^44,53,55,58,59^ | ^44,49,50^ | ^49,55,60^ |
| 2022 | ^64–75^ | ^64–67,69–71,73–77^ | ^64,66–68,72^ | ^66^ | ^66,72,73^ | ^73^ |
| 2023 | ^78–89^ | ^78–81,83–95^ | ^85,86^ | ^78,85,88^ | ^82,86,88,89^ | ^88,89,93,94^ |
| 2024 | ^96–102^ | ^96–98,100,101,103–107^ | ^99^ | ^99,101^ | NA | NA |
| 2025 | ^108–113^ | ^108,110,112–120^ | ^111,113,120,121^ | NA | ^111^ | ^114,116,117,119,120^ |
| 2026 | ^122^ | ^123–130^ | ^127,131^ | ^127^ | ^122,124,131^ | ^124,127,128^ |

Reference

1.	Wang, Y. *et al.* Implementation of Intraoperative Neuromonitoring for Transoral Endoscopic Thyroid Surgery: A Preliminary Report. *J. Laparoendosc. Adv. Surg. Tech. A* **26**, 965–971 (2016).

2.	Anuwong, A. Transoral Endoscopic Thyroidectomy Vestibular Approach: A Series of the First 60 Human Cases. *World J. Surg.* **40**, 491–497 (2016).

3.	Jitpratoom, P., Ketwong, K., Sasanakietkul, T. & Anuwong, A. Transoral endoscopic thyroidectomy vestibular approach (TOETVA) for Graves’ disease: a comparison of surgical results with open thyroidectomy. *Gland Surg.* **5**, 546–552 (2016).

4.	Park, J.-O. & Sun, D.-I. Transoral endoscopic thyroidectomy: our initial experience using a new endoscopic technique. *Surg. Endosc. Interv. Tech.* **31**, 5436–5443 (2017).

5.	Russell, J. O. *et al.* Transoral thyroidectomy and parathyroidectomy - A North American series of robotic and endoscopic transoral approaches to the central neck. *Oral Oncol.* **71**, 75–80 (2017).

6.	Dionigi, G. *et al.* Transoral endoscopic thyroidectomy: preliminary experience in Italy. *Updat. Surg.* **69**, 225–234 (2017).

7.	Wu, Y.-J. *et al.* What is the Appropriate Nodular Diameter in Thyroid Cancer for Extraction by Transoral Endoscopic Thyroidectomy Vestibular Approach Without Breaking the Specimens? A Surgicopathologic Study. *Surg. Laparosc. Endosc. Percutan. Tech.* **28**, 390–393 (2018).

8.	Tesseroli, M. A. S., Spagnol, M. & Sanabria, Á. Transoral endoscopic thyroidectomy by vestibular approach (TOETVA): initial experience in Brazil. *Rev. Col. Bras. Cir.* **45**, e1951 (2018).

9.	Russell, J. O. *et al.* Anterior cervical incision-sparing thyroidectomy: Comparing retroauricular and transoral approaches. *Laryngoscope Investig. Otolaryngol.* **3**, 409–414 (2018).

10.	Wang, Y. *et al.* Transoral endoscopic thyroid surgery via the tri-vestibular approach with a hybrid space-maintaining method: A preliminary report. *Head Neck* **40**, 1774–1779 (2018).

11.	Bakkar, S., Al Hyari, M., Naghawi, M., Corsini, C. & Miccoli, P. Transoral thyroidectomy: a viable surgical option with unprecedented complications-a case series. *J. Endocrinol. Invest.* **41**, 809–813 (2018).

12.	Sivakumar, T. & Amizhthu, R. A. Transoral endoscopic total thyroidectomy vestibular approach: A case series and literature review. *J. Minimal Access Surg.* **14**, 118–123 (2018).

13.	Fu, J. *et al.* Transoral Endoscopic Thyroidectomy: Review of 81 Cases in a Single Institute. *J. Laparoendosc. Adv. Surg. Tech.* **28**, 286–291 (2018).

14.	Chen, H.-K. *et al.* Application of transoral continuous intraoperative neuromonitoring in natural orifice transluminal endoscopic surgery for thyroid disease: a preliminary study. *Surg. Endosc.* **32**, 517–525 (2018).

15.	Yi, J. W. *et al.* Transoral endoscopic surgery for papillary thyroid carcinoma: initial experiences of a single surgeon in South Korea. *Ann. Surg. Treat. Res.* **95**, 73–79 (2018).

16.	Anuwong, A. *et al.* Transoral endoscopic thyroidectomy vestibular approach (TOETVA): indications, techniques and results. *Surg. Endosc.* **32**, 456–465 (2018).

17.	Pérez-Soto, R. H. *et al.* Transoral Endoscopic Thyroidectomy by Vestibular Approach-Initial Experience and Comparative Analysis in the First Reported Mexican Cohort. *J. Laparoendosc. Adv. Surg. Tech. A* **29**, 1526–1531 (2019).

18.	Zhang, D. *et al.* Classifying pain in transoral endoscopic thyroidectomy. *J. Endocrinol. Invest.* **42**, 1345–1351 (2019).

19.	Park, J.-O. *et al.* Gasless transoral endoscopic thyroidectomy vestibular approach (gasless TOETVA). *Surg. Endosc.* **33**, 3034–3039 (2019).

20.	Park, J.-O., Anuwong, A., Kim, M. R., Sun, D.-I. & Kim, M.-S. Transoral endoscopic thyroid surgery in a Korean population. *Surg. Endosc.* **33**, 2104–2113 (2019).

21.	Kadem, S. G., Habash, S. M. & Jasim, A. H. Transoral Endoscopic Thyroidectomy via Vestibular Approach: A series of the first ten cases in Iraq. *Sultan Qaboos Univ. Med. J.* **19**, e68–e72 (2019).

22.	Kim, H. K. *et al.* Transoral Robotic Thyroidectomy for Papillary Thyroid Carcinoma: Perioperative Outcomes of 100 Consecutive Patients. *WORLD J. Surg.* **43**, 1038–1046 (2019).

23.	Han, S. *et al.* Functional Voice and Swallowing Outcome Analysis After Thyroid Lobectomy: Transoral Endoscopic Vestibular Versus Open Approach. *World J. Surg.* **44**, 4127–4135 (2020).

24.	Dinç, B., İlker Turan, M., Rıza Gündüz, U. & Haluk Belen, N. Transoral endoscopic thyroidectomy vestibular approach (TOETVA): Our outcomes from Turkey. *Turk. J. Surg.* **36**, 340–346 (2020).

25.	Zhang, G. *et al.* Pros and Cons of Transoral Endoscopic Thyroidectomy Via Vestibular Approach: A Comparative Study. *Surg. Laparosc. Endosc. Percutan. Tech.* **31**, 331–336 (2020).

26.	Tae, K. *et al.* Sensory change in the chin and neck after transoral thyroidectomy: Prospective study of mental nerve injury. *Head Neck* **42**, 3111–3117 (2020).

27.	Fernandez-Ranvier, G. *et al.* Transoral Endoscopic Thyroidectomy Vestibular Approach: A Single-institution Experience of the First 50 Cases. *Surg. Innov.* **27**, 439–444 (2020).

28.	Dinc, B., Gunduz, U. R. & Belen, N. H. Transoral Endoscopic Thyroidectomy Vestibular Approach (TOETVA) in Thyroiditis. *Surg. Laparosc. Endosc. Percutan. Tech.* **31**, 188–192 (2020).

29.	Hong, Y. T., Ahn, J.-H., Kim, J. H., Yi, J. W. & Hong, K. H. Bi-institutional experience of transoral endoscopic thyroidectomy: Challenges and outcomes. *Head Neck* **42**, 2115–2122 (2020).

30.	Bhargav, P. R. K., Sabaretnam, M. & Amar, V. Endoscopic Thyroidectomy Through Oro-Vestibular Route (ETOVR). *INDIAN J. Surg.* **82**, 598–603 (2020).

31.	Le, Q. V., Ngo, D. Q., Tran, T. D. & Ngo, Q. X. Transoral Endoscopic Thyroidectomy Vestibular Approach: An Initial Experience in Vietnam. *Surg. Laparosc. Endosc. Percutan. Tech.* **30**, 209–213 (2020).

32.	Yi, J. W., Kim, S. -j. & Lee, K. E. Evaluation of the efficacy of postoperative antibiotic treatment in transoral endoscopic thyroidectomy: a prospective randomised controlled trial. *Br. J. ORAL Maxillofac. Surg.* **58**, 334–340 (2020).

33.	Ahn, J.-H., Kim, J. H., Yi, J. W. & Hur, M. H. Comparison between the 4K ultra-high definition (UHD) and high definition (HD) endoscopic systems for transoral endoscopic thyroidectomy. *Gland Surg.* **9**, 229–237 (2020).

34.	Fernandez Ranvier, G. *et al.* International Multi-institutional Experience with the Transoral Endoscopic Thyroidectomy Vestibular Approach. *J. Laparoendosc. Adv. Surg. Tech. A* **30**, 278–283 (2020).

35.	Luo, J.-H., Xiang, C., Wang, P. & Wang, Y. The Learning Curve for Transoral Endoscopic Thyroid Surgery: A Single Surgeon’s 204 Case Experience. *J. Laparoendosc. Adv. Surg. Tech. A* **30**, 163–169 (2020).

36.	Peng, X. *et al.* The clinical application of mental nerve dissection in transoral endoscopic thyroidectomy via an oral vestibular approach. *Surg. Endosc.* **34**, 153–158 (2020).

37.	Kim, M. *et al.* Can voice pitch be preserved in patients after transoral endoscopic thyroidectomy vestibular approach? *J. Clin. Med.* **9**, 1‐11 (2020).

38.	Fu, Y. *et al.* TransOral Endoscopic Thyroidectomy via Submental and Vestibular Approach: A Preliminary Report. *Front. Surg.* **7**, 591522 (2020).

39.	Tan, Y. *et al.* Transoral endoscopic selective lateral neck dissection for papillary thyroid carcinoma: a pilot study. *Surg. Endosc.* **34**, 5274–5282 (2020).

40.	Wang, T. *et al.* Safety of central compartment neck dissection for transoral endoscopic thyroid surgery in papillary thyroid carcinoma. *Jpn. J. Clin. Oncol.* **50**, 387–391 (2020).

41.	Sun, H. *et al.* Comparison of transoral endoscopic thyroidectomy vestibular approach, total endoscopic thyroidectomy via areola approach, and conventional open thyroidectomy: a retrospective analysis of safety, trauma, and feasibility of central neck dissection in the treatment of papillary thyroid carcinoma. *Surg. Endosc.* **34**, 268–274 (2020).

42.	Guo, F. *et al.* Comparative Study Between Endoscopic Thyroid Surgery via the Oral Vestibular Approach and the Areola Approach. *J. Laparoendosc. Adv. Surg. Tech. A* **30**, 170–174 (2020).

43.	Yeh, Y.-T. *et al.* Printing a Three-Dimensional Patient-Specific Safety Device for Reducing the Potential Risk of Mental Nerve Injury During Transoral Thyroidectomy. *World J. Surg.* **44**, 371–377 (2020).

44.	Alsafran, S. *et al.* Trans-oral endoscopic endocrine surgery vestibular approach: Pioneering the technique in the Gulf Cooperation Council Countries - A case series. *Ann. Med. Surg.* **72**, (2021).

45.	Chen, Y.-H., Kim, H.-Y., Anuwong, A., Huang, T.-S. & Duh, Q.-Y. Transoral robotic thyroidectomy versus transoral endoscopic thyroidectomy: a propensity-score-matched analysis of surgical outcomes. *Surg. Endosc.* **35**, 6179–6189 (2021).

46.	Wongwattana, P., Laoveerakul, P. & Santeerapharp, A. A comparison of efficacy and quality of life between transoral endoscopic thyroidectomy vestibular approach (TOETVA) and endoscopic thyroidectomy axillo-breast approach (ETABA) in thyroid surgery: non-randomized clinical trial. *Eur. Arch. Oto-Rhino-Laryngol. Off. J. Eur. Fed. Oto-Rhino-Laryngol. Soc. EUFOS Affil. Ger. Soc. Oto-Rhino-Laryngol. - Head Neck Surg.* **278**, 4043–4049 (2021).

47.	Park, J.-O. *et al.* Transoral endoscopic thyroidectomy using a self-retaining retractor as an alternative to carbon dioxide gas insufflation: A comparative analysis of 131 cases. *Oral Oncol.* **121**, 105463 (2021).

48.	Shen, S. *et al.* Comparing quality of life between patients undergoing trans-areola endoscopic thyroid surgery and trans-oral endoscopic thyroid surgery. *BMC Surg.* **21**, 277 (2021).

49.	Zheng, G. *et al.* Safety and surgical outcomes of transoral endoscopic thyroidectomy vestibular approach for papillary thyroid cancer: A two-centre study. *Eur. J. Surg. Oncol. J. Eur. Soc. Surg. Oncol. Br. Assoc. Surg. Oncol.* **47**, 1346–1351 (2021).

50.	Chand, G. *et al.* Natural Orifice Endoscopic Thyroidectomy via Transoral Vestibular Approach (TOVA): Single Surgeon Experience from North India. *Indian J. Otolaryngol. Head Neck Surg. Off. Publ. Assoc. Otolaryngol. India* **73**, 160–166 (2021).

51.	Perigli, G. *et al.* An easier option for ‘invisible scar’ thyroidectomy: hybrid-transoral endoscopic thyroidectomy submental access (H-TOETSA)-experience on twenty-two consecutive patients. *Surg. Endosc.* **35**, 1796–1800 (2021).

52.	Deroide, G. *et al.* Trans oral endoscopic thyroidectomy (TOETVA): First French experience in 90 patients. *J. Visc. Surg.* **158**, 103–110 (2021).

53.	Razavi, C. R. *et al.* The variable direct cost and cost drivers of transoral endoscopic thyroidectomy vestibular approach. *Gland Surg.* **10**, 521–528 (2021).

54.	Khafif, A. *et al.* Adoption of the transoral endoscopic vestibular approach by head and neck surgeons without prior laparoscopic/robotic experience. *Head Neck* **43**, 496–504 (2021).

55.	Ngo, D. Q., Le, D. T., Hoang, G. N., Ngo, Q. X. & Le, Q. V. Case Report: Transoral Endoscopic Thyroidectomy via Vestibular Approach in Pediatric Thyroid Cancer. *Front. Pediatr.* **9**, 765278 (2021).

56.	Liu, Z. *et al.* Postoperative drain after transoral endoscopic thyroidectomy vestibular approach (TOETVA) with single incision. *Surg. Endosc.* **35**, 358‐366 (2021).

57.	Kumar, C. *et al.* Endoscopic thyroidectomy: which one is the better technique for the beginners? *J. Minimal Access Surg.* **17**, 337‐341 (2021).

58.	Kose, O. C., Turk, Y., Ozdemir, M., Makay, O. & Icoz, G. Patient Eligibility for Transoral Endoscopic Thyroidectomy Vestibular Approach in an Endemic Region. *Sisli Etfal Hastan. Tip Bul.* **55**, 304–309 (2021).

59.	G., F.-R. G. *et al.* Transoral Endoscopic Thyroidectomy Vestibular Approach (TOETVA): Treatment for Benign Thyroid Diseases. *J. Endocr. Surg.* **21**, 13–19 (2021).

60.	Zheng, G. *et al.* The sensorimotor changes of the lower lip and chin after transoral endoscopic thyroidectomy vestibular approach. *Updat. Surg.* **73**, 2283–2291 (2021).

61.	Liu, Z. *et al.* Comparison of the transoral endoscopic thyroidectomy vestibular approach and open thyroidectomy: A propensity score-matched analysis of surgical outcomes and safety in the treatment of papillary thyroid carcinoma. *Surgery* **170**, 1680–1686 (2021).

62.	Zhang, W.-D. *et al.* Transoral Endoscopic Thyroidectomy Vestibular Approach Versus Endoscopic Thyroidectomy Via Areola Approach for Patients With Unilateral Papillary Thyroid Carcinoma: A Retrospective Study. *Surg. Laparosc. Endosc. Percutan. Tech.* **31**, 550–553 (2021).

63.	Chai, Y. J., Chae, S., Oh, M. Y., Kwon, H. & Park, W. S. Transoral Endoscopic Thyroidectomy Vestibular Approach (TOETVA): Surgical Outcomes and Learning Curve. *J. Clin. Med.* **10**, (2021).

64.	Van Den Heede, K., Brusselaers, N., Gaujoux, S., Menegaux, F. & Chereau, N. Feasibility and Safety of Ambulatory Transoral Endoscopic Thyroidectomy via Vestibular Approach (TOETVA). *World J. Surg.* **46**, 2678–2686 (2022).

65.	Chen, H., Deng, L., Xu, K., Gong, Z. & Zhu, X. Clinical application of transoral and submental thyroidectomy (TOaST): a series of 54 human cases. *LANGENBECKS Arch. Surg.* **407**, 3039–3044 (2022).

66.	Jabbar, S. A. & Alla Werdi, N. I. Transoral endoscopic thyroidectomy vestibular approach (TOETVA): first twelve case series in Erbil, Iraq. *J. Med. Life* **15**, 1283–1293 (2022).

67.	Shah, S. Transoral Endoscopic Thyroidectomy Vestibular Approach (TOETVA)-a Case Series Analysis. *INDIAN J. Surg.* https://doi.org/10.1007/s12262-022-03558-2 (2022) doi:10.1007/s12262-022-03558-2.

68.	Muslim, S. A SINGLE SURGEON’S EXPERIENCE IN DEFINING THE LEARNING CURVE FOR TRANSORAL ENDOSCOPIC THYROIDECTOMY -VESTIBULAR APPROACH (TOETVA). *Georgian Med. News* 59–64 (2022).

69.	Lee, M.-C., Ahn, J., Choi, I. J., Lee, B.-C. & Ryu, J. Comparative Study of Gasless Transaxillary, Bilateral Axillo-Breast, Unilateral Axillo-Breast With Carbon Dioxide Insufflation, Retroauricular, and Transoral Vestibular Endoscopic Thyroidectomy Approaches at a Single Institution: A Retrospective Analysis and Lessons Learned. *Clin. Exp. Otorhinolaryngol.* **15**, 283–291 (2022).

70.	Nguyen, H. X., Nguyen, H. X., Nguyen, T. T. P. & Van Le, Q. Transoral endoscopic thyroidectomy by vestibular approach in Viet Nam: surgical outcomes and long-term follow-up. *Surg. Endosc.* **36**, 4248–4254 (2022).

71.	Cohen, O. *et al.* Trans-oral endoscopic thyroidectomy vestibular approach (TOETVA) for the pediatric population: a multicenter, large case series. *Surg. Endosc.* **36**, 2507–2513 (2022).

72.	Nguyen, H. X., Nguyen, H. X., Le, A. D. & Van Le, Q. Comparison of Transoral Endoscopic Thyroidectomy Vestibular Approach and Conventional Open Thyroidectomy in Benign Thyroid Tumors. *Indian J. Surg. Oncol.* **13**, 178–183 (2022).

73.	Zheng, G. *et al.* Transoral versus gasless transaxillary endoscopic thyroidectomy: a comparative study. *Updat. Surg.* **74**, 295–302 (2022).

74.	Xuan Nguyen, H., Nguyen, H. X., Thi Hoang, H. & Van Le, Q. Quality of Life and Surgical Outcome of Transoral Endoscopic Thyroidectomy Vestibular Approach (TOETVA) versus Open Thyroid Surgery: Experience from a Single Center in Vietnam. *J. Thyroid Res.* **2022**, 2381063 (2022).

75.	Sun, H. *et al.* Comparison Between Transoral Endoscopic Thyroidectomy Vestibular Approach (TOETVA) and Conventional Open Thyroidectomy for Patients Undergoing Total Thyroidectomy and Central Neck Dissection: A Propensity Score-Matching Analysis. *Front. Oncol.* **12**, 856021 (2022).

76.	Chen, H., Deng, L., Gong, Z. & Zhu, X. The Advantages of Applying a 5-mm Endoscope in the Transoral Endoscopic Thyroidectomy Vestibular Approach. *Surg. Laparosc. Endosc. Percutan. Tech.* **32**, 357–361 (2022).

77.	Yan, X. *et al.* Transoral endoscopic thyroidectomy vestibular approach for papillary thyroid microcarcinoma: an analysis of clinical outcomes. *Am. J. Transl. Res.* **14**, 7907–7915 (2022).

78.	Jin, X., Huang, Z., Guo, P. & Yuan, R. TOETVA: a single surgeon’s learning curve and a case report of CASTLE thyroid tumor. *Langenbecks Arch. Surg.* **408**, 398 (2023).

79.	Ma, T. *et al.* Transoral and submental endoscopic thyroidectomy (TOaST) for early stage papillary thyroid carcinoma: a real-world data retrospective cohort study. *Surg. Endosc.* **37**, 7649–7657 (2023).

80.	Li, Y. *et al.* Is transoral endoscopic thyroidectomy safe for total thyroidectomy compared to open thyroidectomy? A propensity-score matched cohort study with papillary thyroid carcinoma. *J. Surg. Oncol.* **128**, 502–509 (2023).

81.	Kim, S., Kim, G.-W. & Hong, Y. T. Phonetic features of patients with transoral endoscopic thyroidectomy. *Auris. Nasus. Larynx* **50**, 593–600 (2023).

82.	Bhandarwar, A. *et al.* Endoscopic Thyroidectomy: A 6-Year Experience from a Tertiary Care Teaching Hospital in Western India. *J. Laparoendosc. Adv. Surg. Tech. A* **33**, 728–737 (2023).

83.	Pradhan, S., Fernando, D. T., Tien, A., Wong, S. L. & Yew, M. K. Transoral endoscopic thyroidectomy vestibular approach (TOETVA): initial experience in Australia. *ANZ J. Surg.* **93**, 545–549 (2023).

84.	Lee, M. J., Oh, M. Y., Lee, J.-M., Sun, J. & Chai, Y. J. Comparative surgical outcomes of transoral endoscopic and robotic thyroidectomy for thyroid carcinoma: a propensity score-matched analysis. *Surg. Endosc.* **37**, 1132–1139 (2023).

85.	Llorente, P. M. *et al.* Transoral endoscopic thyroidectomy vestibular approach: Results after 53 first cases. *CIRUGIA ESPANOLA* **101**, 35–42 (2023).

86.	Ünlü, M. T., Aygun, N., Serin, E. & Uludag, M. Comparison of transoral endoscopic thyroidectomy vestibular approach and open conventional thyroidectomy regardıng inflammatory responses, pain, and patient satisfaction: a prospective study. *Front. Surg.* **10**, 1281093 (2023).

87.	Sun, H., Chu, Y., Zhang, G., Zheng, G. & Zheng, H. The ambulatory transoral endoscopic thyroidectomy vestibular approach is safe and economical for patients with thyroid nodules. *Front. Endocrinol.* **14**, 1116280 (2023).

88.	Ngo, D. Q., Le, D. T., Ngo, Q. X. & Van Le, Q. Transoral endoscopic thyroidectomy vestibular approach as a novel technique for pediatric populations: Results from a single surgeon. *Front. Endocrinol.* **14**, 1177633 (2023).

89.	Jiang, J. *et al.* Gasless submental-transoral combined approach endoscopic thyroidectomy: a new surgical technique. *Front. Oncol.* **13**, 1115927 (2023).

90.	Zhang, W.-D. *et al.* Transoral Endoscopic Thyroidectomy Vestibular Approach Versus Gasless Transaxillary Endoscopic Thyroidectomy for Patients With Unilateral Papillary Thyroid Carcinoma: A Retrospective Study. *Surg. Laparosc. Endosc. Percutan. Tech.* **33**, 347–350 (2023).

91.	Nguyen, K. A. & Nguyen, N. D. Transoral endoscopic thyroidectomy with or without central neck dissection. *Am. J. Otolaryngol.* **44**, 103728 (2023).

92.	Lee, J. H., Choi, H. J., Woo, J.-W. & Jung, E.-J. Robotic versus endoscopic transoral thyroidectomy in papillary thyroid cancer: A comparative analysis of surgical outcomes in 240 consecutive patients. *Head Neck* **45**, 827–837 (2023).

93.	Jiang, J. *et al.* Novel suspension system for gasless transoral vestibular thyroidectomy. *Surg. Endosc.* **37**, 1070–1076 (2023).

94.	Xuan, H. N., Anh, T. D., Xuan, H. N., Thai, D. P. & Le Van, Q. Occult Central Lymph Node Metastasis in cN0 Papillary Thyroid Carcinoma Patients Undergoing TOETVA Procedure. *J. Thyroid Res.* **2023**, 4779409 (2023).

95.	Wang, Y., Zhang, L., Huang, J. & Wang, L. Preoperative application of carbon nanoparticles in transoral endoscopic thyroidectomy vestibular approach for papillary thyroid cancer. *Front. Oncol.* **13**, 1120411 (2023).

96.	Wen, X. *et al.* ‘Three-in-One Wonder’: A Retrospective Cohort Study on Modified Robotic-Assisted Transoral Thyroidectomy. *J. Otolaryngol. - Head Neck Surg. J. Oto-Rhino-Laryngol. Chir. Cervico-Faciale* **53**, 19160216241304384 (2024).

97.	Harsha, M. P., Padha, K., Dwivedi, D. & Mateen, M. A. Natural Orifice Trans-oral, Trans-vestibular Endoscopic Thyroidectomy (NO-TOTVET): a Case Series. *Indian J. Surg. Oncol.* **15**, 721–725 (2024).

98.	Liang, T.-J., Chen, I.-S., Chen, Y.-C. & Liu, S.-I. Feasibility of completion thyroidectomy via transoral endoscopic vestibular approach. *Updat. Surg.* **76**, 2565–2571 (2024).

99.	Ngo, Q. X. *et al.* Transoral endoscopic thyroidectomy vestibular approach versus conventional open thyroidectomy for the treatment of benign thyroid tumours: A prospective cohort study. *J. Minimal Access Surg.* **20**, 403–407 (2024).

100.	Wang, S.-H. *et al.* Application and advantages of a joystick-controlled robotic scope holder in transoral endoscopic thyroidectomy vestibular approach: a comparative study. *Updat. Surg.* **76**, 1425–1434 (2024).

101.	Nagururu, N. V. *et al.* Long-term Quality of Life After Thyroidectomy: Transoral Endoscopic Thyroidectomy Vestibular Approach Versus Transcervical Approach. *Otolaryngol.--Head Neck Surg. Off. J. Am. Acad. Otolaryngol.-Head Neck Surg.* **171**, 45–53 (2024).

102.	Kumar, A., Dhar, A., Srivastava, A. & Kumar, R. A Prospective Comparative Analysis to Study the Impact on Voice Changes Following Endoscopic Thyroidectomy. *Surg. Laparosc. Endosc. Percutan. Tech.* **34**, 407‐412 (2024).

103.	Zhou, S. *et al.* Defining the Thyroid-RLN Entry Triangle for Enhanced Recurrent Laryngeal Nerve Exposure in TOETVA: A Retrospective Study. *J. Otolaryngol. - Head Neck Surg. J. Oto-Rhino-Laryngol. Chir. Cervico-Faciale* **53**, 19160216241301328 (2024).

104.	Zhang, W.-D., Le, Q., Yu, K.-J., Wang, Y.-C. & Wu, X.-J. Application of Carbon Nanoparticles in Transoral Endoscopic Thyroidectomy Vestibular Approach for Unilateral Papillary Thyroid Cancer. *Surg. Laparosc. Endosc. Percutan. Tech.* **34**, 301–305 (2024).

105.	Li, N. *et al.* A New Transoral Vestibulum Single Incision Endoscopic-Assisted Thyroidectomy with Gasless. *The Laryngoscope* **134**, 2976–2984 (2024).

106.	Qian, L. *et al.* The impact of body mass index on operative time in transoral endoscopic thyroidectomy vestibular approach for thyroid cancer. *Endocrine* **84**, 179–184 (2024).

107.	Deng, Y. *et al.* Transoral endoscopic thyroid lobectomy and ipsilateral central neck lymph node dissection vestibular approach: analysis of the learning curve and clinical outcomes evaluation. *Front. Endocrinol.* **15**, 1498797 (2024).

108.	Wu, B., Chen, X. & Huang, J. Antibiotic Use Can be De-escalated During Transoral Endoscopic Thyroidectomy: A Bacterial Culture-Based Study. *Ear. Nose. Throat J.* **104**, NP669–NP674 (2025).

109.	Yadav, S. K., Johri, G., Shekhar, S., Agarwal, P. & Sharma, D. The Learning Curve for Transoral Endoscopic Thyroidectomy Without Neuromonitoring: Analysis of First 103 Cases From India. *OTO Open* **9**, e70142 (2025).

110.	Uen, Y.-H., Wu, C.-W., Wen, K.-S., Lin, Y.-F. & Lin, K.-Y. Percutaneous continuous vagal stimulation provided superior neuromonitoring over the peroral electrode in transoral thyroidectomy. *Surg. Endosc.* **39**, 6249–6258 (2025).

111.	Wang, M. *et al.* Transoral vestibular robotic thyroidectomy in pediatric thyroid disease: 5 case reports. *Endocrine* **88**, 523–528 (2025).

112.	Chand, G., Godi, P., Kumari, M. & Yadav, A. Trans-Oral Endoscopic Thyroidectomy via Vestibular Approach (TOETVA): Single-Surgeon Experience of more than 100 Cases. *INDIAN J. Surg.* https://doi.org/10.1007/s12262-025-04302-2 (2025) doi:10.1007/s12262-025-04302-2.

113.	Zhang, D., Wan, L., Frattini, F. & Dionigi, G. Increased acute-phase reaction after the transoral endoscopic thyroidectomy vestibular approach (TOEVA) compared with minimally invasive video-assisted thyroidectomy (MIVAT) for unilateral thyroidectomy. *Surg. Today* **55**, 969‐976 (2025).

114.	Zhang, X. *et al.* Comparison of Transoral Endoscopic Thyroidectomy via the Vestibular Approach and Conventional Open Thyroidectomy for Papillary Thyroid Carcinoma: A Propensity Score-Matched Analysis. *Ann. Surg. Oncol.* **32**, 6765–6773 (2025).

115.	Li, H., Liu, Z. & Li, W. The central lymph node dissection using carbon nanoparticle in transoral vestibular approach endoscopic thyroid surgery. *Sci. Rep.* **15**, 28320 (2025).

116.	Ngo, D. Q., Truong, D. S., Le, D. T. & Hoang, T. M. Transoral endoscopic thyroidectomy vestibular approach for pT1b papillary thyroid carcinoma: surgical and oncological outcomes. *Oral Oncol.* **167**, 107406 (2025).

117.	Nguyen, H. X., Nguyen, H. X., Nguyen, T. N., Luu, L. H. & Le, Q. V. Male Thyroid Cancer Patients Treated by Transoral Endoscopic Thyroidectomy Vestibular Approach (TOETVA): Surgical Outcomes and Distinct Experiences. *INDIAN J. Surg. Oncol.* https://doi.org/10.1007/s13193-025-02284-x (2025) doi:10.1007/s13193-025-02284-x.

118.	Deng, Z., Hu, X., Zeng, F., Guo, Y. & Peng, X. Determining the optimal port placement for transoral endoscopic thyroidectomy vestibular approach in a retrospective study. *Sci. Rep.* **15**, 6948 (2025).

119.	Sheng, X., Fang, J., Chen, G., Li, W. & Wang, S. A suspension surgical space construction method involving a gasless transoral endoscopic thyroidectomy vestibular approach: a single-center experience. *World J. Surg. Oncol.* **23**, 58 (2025).

120.	Zhou, J., Li, X., Xia, F. & Su, D. Enhanced cosmetic outcomes with central venous catheter drainage in transoral vestibular endoscopic thyroidectomy: a prospective randomized-controlled trial. *J. Robot. Surg.* **19**, 498 (2025).

121.	Unlu, M. *et al.* Did scarless thyroidectomy meet expectations? An evaluation in the aspect of cosmesis: a single-centre prospective study. *J. Minimal Access Surg.* https://doi.org/10.4103/jmas.jmas_27_25 (2025) doi:10.4103/jmas.jmas_27_25.

122.	Rege, S. A., Vasa, D., Shah, S. & Salvi, V. Endoscopic thyroidectomy - Is it safe and reliable? - Experience and results at a tertiary care centre in India. *J. Minimal Access Surg.* https://doi.org/10.4103/jmas.jmas_309_25 (2026) doi:10.4103/jmas.jmas_309_25.

123.	Zhang, Y., Hu, H., Tan, J.-T., Yao, T.-J. & Qian, L.-Y. A comparative study of transoral and submental versus vestibular endoscopic thyroidectomy in patients with papillary thyroid carcinoma. *Surg. Endosc.* **40**, 512–518 (2026).

124.	Zhang, D., Brucchi, F., Colombo, C. & Dionigi, G. Ten years after transoral endoscopic thyroidectomy vestibular approach (TOETVA): Why did we abandon the transoral route? *Surgery* **196**, 110292 (2026).

125.	Xu, T. *et al.* Surgical efficiency, safety, and quality of life among four various approaches for T1 stage papillary thyroid carcinoma: a prospective cohort study. *Surg. Endosc.* **40**, 1984–1998 (2026).

126.	Lee, J. H. & Gwak, H. Oncologic adequacy and outcomes of open, transoral endoscopic, and robotic thyroidectomy for papillary thyroid carcinoma: a propensity score-matched analysis. *World J. Surg. Oncol.* **24**, 5 (2025).

127.	Ngo, D. Q., Le, D. T., Ngo, Q. X. & Le, Q. V. Surgical outcomes of modified versus conventional transoral endoscopic thyroidectomy vestibular approach for papillary thyroid carcinoma. *Oral Oncol.* **175**, 107908 (2026).

128.	Ngo, D. Q. *et al.* Technical and therapeutic evaluation of endoscopic treatment of early papillary thyroid cancer through oral vestibular approach. *Oral Oncol.* **180**, 108061 (2026).

129.	Qin, J. *et al.* Comparison of surgical trauma between three endoscopic thyroidectomy approaches and open surgery. *Front. Oncol.* **15**, 1677239 (2025).

130.	Nguyen, H. X., Nguyen, H. X., Nguyen, T. N., Luu, L. H. & Van Le, Q. Male Thyroid Cancer Patients Treated by Transoral Endoscopic Thyroidectomy Vestibular Approach (TOETVA): Surgical Outcomes and Distinct Experiences. *Indian J. Surg. Oncol.* **16**, 1738–1744 (2025).

131.	Unlu, M. T. *et al.* Did scarless thyroidectomy meet expectations? An evaluation in the aspect of cosmesis: A single-centre prospective study. *J. MINIMAL ACCESS Surg.* **22**, 51–56 (2026).
