# Supplementary material for: Preliminary experience with the transoral endoscopic thyroidectomy vestibular approach in selected complex cases: a case series and literature review
Source: Front Surg. 2026 Jul 27;13:1882842. doi: 10.3389/fsurg.2026.1882842 (PMC13454321; doi:10.3389/fsurg.2026.1882842)
Supplement: Supplementary file 3 [file Table2.docx]

**Supplementary Table 2**. Summary of TOETVA Inclusion Criteria by Country/Region and Year

| Country (No. of Studies) | Year | Benign Nodule Size (cm) | Malignant Tumor Size (cm) | Thyroid Volume (ml) | Thyroid Diameter (cm) |
| --- | --- | --- | --- | --- | --- |
| China (48) |  |  |  |  |  |
|  | 2015 | ≤5^1^/6^2^ | ≤2^2^ | NA | ≤8^3^ |
|  | 2016 | NA | ≤3^4^ | NA | NA |
|  | 2017 | ≤3.5^5^/6^6^ | ≤1^5^/3^6^ | ≤25^5^ | ≤10^5^ |
|  | 2018 | ≤5^7^/6^8,9^ | ≤2^8–11^/3^12^ | NA | NA |
|  | 2019 | ≤5^13^/6^14^ | ≤1^15^/3^14^/4^16^ | NA | NA |
|  | 2020 | ≤6^17,18^ | ≤1^19^/2^17,20–22^/4^18^ | NA | NA |
|  | 2021 | ≤4^23,24^/6^25–27^ | ≤1^28^/2^23,24,26,29–31^/3^25,27^ | NA | NA |
|  | 2022 | ≤3.5^32^/6^33,34^ | ≤1^32^/2^35–37^/3^33^/4^34^ | ≤25^32^ | NA |
|  | 2023 | ≤6^38,39^ | ≤2^40,41^/3^38,42,43^ | ≤50^39^ | ≤10^38^ |
|  | 2024 | ≤4^44^ | ≤2^44–48^ | ≤50^45^ | NA |
| Korea (18) |  |  |  |  |  |
|  | 2017 | ≤8^49^ | ≤2^50^/2.5^49^/3^51^ | NA | NA |
|  | 2018 | ≤8^52–54^ | ≤2^52–54^ | NA | NA |
|  | 2019 | ≤4^55^/5^56^/6^57^/8^58,59^ | ≤2^55,59^/2.5^58^/3^56,57^ | NA | NA |
|  | 2020 | NA | ≤4^60^ | NA | NA |
|  | 2021 | ≤6^61,62^/8^63^ | ≤2^61,63^/3^62^ | NA | NA |
|  | 2022 | ≤4^64^ | ≤2^64,65^ | NA | NA |
|  | 2023 | NA | ≤2^66^ | NA | NA |
| Vietnam (14) |  |  |  |  |  |
|  | 2019 | ≤6^67^ | ≤2^67^ | NA | ≤10^67^ |
|  | 2020 | ≤6^68^ | ≤2^68^ | NA | ≤10^68^ |
|  | 2021 | ≤6^69–72^ | ≤2^69,71,73,74^ | ≤10^70^/45^72^ | ≤10^72^ |
|  | 2022 | ≤6^75^ | ≤2^75^ | NA | ≤10^75^ |
|  | 2023 | NA | ≤2^76,77^ | NA | NA |
|  | 2024 | NA | ≤2^78^ | NA | NA |
|  | 2025 | NA | ≤1^79^/≤2^80^ | ≤45^79^ | ≤10^79^ |
| India (10) |  |  |  |  |  |
|  | 2016 | ≤4^81^ | NA | ≤30^81^ | NA |
|  | 2018 | ≤3^82^/4^83^ | ≤3^82^ | NA | NA |
|  | 2019 | ≤6^84^ | NA | NA | NA |
|  | 2020 | ≤6^85^ | NA | NA | NA |
|  | 2021 | ≤5^86^ | ≤4^86^ | ≤45^86^ | NA |
|  | 2023 | ≤6^87^ | ≤2^87^ | NA | NA |
|  | 2024 | ≤4^88,89^ | ≤2^88^ | NA | NA |
|  | 2025 | ≤6^90^ | NA | NA | NA |
| United States (7) |  |  |  |  |  |
|  | 2016 | ≤6^91^ | NA | NA | NA |
|  | 2017 | ≤6^92^ | ≤2^92^ | NA | NA |
|  | 2018 | ≤6^93^ | ≤3^93^ | ≤45^93^ | ≤10^93^ |
|  | 2019 | ≤6^94–96^ | ≤2^96^/3^94,95^ | ≤45^94,95^ | ≤10^94–96^ |
|  | 2021 | ≤6^97^ | ≤2^97^ | NA | ≤10^97^ |
| Taiwan (7) |  |  |  |  |  |
|  | 2016 | ≤6^98^ | ≤1^98^ | NA | NA |
|  | 2017 | ≤5^99^ | ≤1^99^ | NA | NA |
|  | 2019 | ≤8^100^ | ≤1^101^/3^100^ | NA | ≤10^101^ |
|  | 2021 | ≤8^102^ | ≤4^102^ | NA | NA |
|  | 2023 | ≤5^103^/6^104^ | ≤1^104^/2^103^ | NA | NA |
| Turkey (6) |  |  |  |  |  |
|  | 2019 | ≤4^105^/6^106^ | ≤2^105,106^ | ≤45^105^ | ≤10^105,106^ |
|  | 2020 | ≤4^107^/6^108^ | ≤1^107^/3^108^ | ≤45^108^/50^107,109^ | ≤10^108^ |
|  | 2021 | NA | NA | ≤50^110^ | NA |

NA = Not available or not reported in the study. When multiple size limits were reported (e.g., 1/2/3 cm), all values are listed to reflect variation among studies. The final year of each study’s enrollment period was used for classification. This table summarizes the inclusion criteria from the top seven highest-publishing countries/regions.

1.	Fu, J. *et al.* Transoral Endoscopic Thyroidectomy: Review of 81 Cases in a Single Institute. *JOURNAL OF LAPAROENDOSCOPIC & ADVANCED SURGICAL TECHNIQUES* **28**, 286–291 (2018).

2.	Wang, Y. *et al.* Transoral endoscopic thyroid surgery via the tri-vestibular approach with a hybrid space-maintaining method: A preliminary report. *Head Neck* **40**, 1774–1779 (2018).

3.	Wang, Y. *et al.* Implementation of Intraoperative Neuromonitoring for Transoral Endoscopic Thyroid Surgery: A Preliminary Report. *J Laparoendosc Adv Surg Tech A* **26**, 965–971 (2016).

4.	Wang, T. *et al.* Safety of central compartment neck dissection for transoral endoscopic thyroid surgery in papillary thyroid carcinoma. *Jpn J Clin Oncol* **50**, 387–391 (2020).

5.	Zhang, D. *et al.* Classifying pain in transoral endoscopic thyroidectomy. *J Endocrinol Invest* **42**, 1345–1351 (2019).

6.	Luo, J.-H., Xiang, C., Wang, P. & Wang, Y. The Learning Curve for Transoral Endoscopic Thyroid Surgery: A Single Surgeon’s 204 Case Experience. *J Laparoendosc Adv Surg Tech A* **30**, 163–169 (2020).

7.	Zhang, G. *et al.* Pros and Cons of Transoral Endoscopic Thyroidectomy Via Vestibular Approach: A Comparative Study. *Surg Laparosc Endosc Percutan Tech* **31**, 331–336 (2020).

8.	Peng, X. *et al.* The clinical application of mental nerve dissection in transoral endoscopic thyroidectomy via an oral vestibular approach. *Surg Endosc* **34**, 153–158 (2020).

9.	Liu, Z. *et al.* Postoperative drain after transoral endoscopic thyroidectomy vestibular approach (TOETVA) with single incision. *Surgical endoscopy* **35**, 358‐366 (2021).

10.	Sun, H. *et al.* Comparison of transoral endoscopic thyroidectomy vestibular approach, total endoscopic thyroidectomy via areola approach, and conventional open thyroidectomy: a retrospective analysis of safety, trauma, and feasibility of central neck dissection in the treatment of papillary thyroid carcinoma. *Surg Endosc* **34**, 268–274 (2020).

11.	Tan, Y. *et al.* Transoral endoscopic selective lateral neck dissection for papillary thyroid carcinoma: a pilot study. *Surg Endosc* **34**, 5274–5282 (2020).

12.	Li, N. *et al.* A New Transoral Vestibulum Single Incision Endoscopic-Assisted Thyroidectomy with Gasless. *Laryngoscope* **134**, 2976–2984 (2024).

13.	Shen, S. *et al.* Comparing quality of life between patients undergoing trans-areola endoscopic thyroid surgery and trans-oral endoscopic thyroid surgery. *BMC Surg* **21**, 277 (2021).

14.	Zheng, G. *et al.* Safety and surgical outcomes of transoral endoscopic thyroidectomy vestibular approach for papillary thyroid cancer: A two-centre study. *Eur J Surg Oncol* **47**, 1346–1351 (2021).

15.	Guo, F. *et al.* Comparative Study Between Endoscopic Thyroid Surgery via the Oral Vestibular Approach and the Areola Approach. *J Laparoendosc Adv Surg Tech A* **30**, 170–174 (2020).

16.	Zheng, G. *et al.* The sensorimotor changes of the lower lip and chin after transoral endoscopic thyroidectomy vestibular approach. *Updates Surg* **73**, 2283–2291 (2021).

17.	Fu, Y. *et al.* TransOral Endoscopic Thyroidectomy via Submental and Vestibular Approach: A Preliminary Report. *Front Surg* **7**, 591522 (2020).

18.	Zheng, G. *et al.* Transoral versus gasless transaxillary endoscopic thyroidectomy: a comparative study. *Updates Surg* **74**, 295–302 (2022).

19.	Liu, Z. *et al.* Comparison of the transoral endoscopic thyroidectomy vestibular approach and open thyroidectomy: A propensity score-matched analysis of surgical outcomes and safety in the treatment of papillary thyroid carcinoma. *Surgery* **170**, 1680–1686 (2021).

20.	Zhang, W.-D. *et al.* Transoral Endoscopic Thyroidectomy Vestibular Approach Versus Endoscopic Thyroidectomy Via Areola Approach for Patients With Unilateral Papillary Thyroid Carcinoma: A Retrospective Study. *Surg Laparosc Endosc Percutan Tech* **31**, 550–553 (2021).

21.	Chen, H., Deng, L., Gong, Z. & Zhu, X. The Advantages of Applying a 5-mm Endoscope in the Transoral Endoscopic Thyroidectomy Vestibular Approach. *Surg Laparosc Endosc Percutan Tech* **32**, 357–361 (2022).

22.	Li, H., Liu, Z. & Li, W. The central lymph node dissection using carbon nanoparticle in transoral vestibular approach endoscopic thyroid surgery. *Sci Rep* **15**, 28320 (2025).

23.	Li, Y. *et al.* Is transoral endoscopic thyroidectomy safe for total thyroidectomy compared to open thyroidectomy? A propensity-score matched cohort study with papillary thyroid carcinoma. *J Surg Oncol* **128**, 502–509 (2023).

24.	Ma, T. *et al.* Transoral and submental endoscopic thyroidectomy (TOaST) for early stage papillary thyroid carcinoma: a real-world data retrospective cohort study. *Surg Endosc* **37**, 7649–7657 (2023).

25.	Sun, H. *et al.* Comparison Between Transoral Endoscopic Thyroidectomy Vestibular Approach (TOETVA) and Conventional Open Thyroidectomy for Patients Undergoing Total Thyroidectomy and Central Neck Dissection: A Propensity Score-Matching Analysis. *Front Oncol* **12**, 856021 (2022).

26.	Chen, H., Deng, L., Xu, K., Gong, Z. & Zhu, X. Clinical application of transoral and submental thyroidectomy (TOaST): a series of 54 human cases. *Langenbecks Arch Surg* **407**, 3039–3044 (2022).

27.	Jiang, J. *et al.* Gasless submental-transoral combined approach endoscopic thyroidectomy: a new surgical technique. *Front Oncol* **13**, 1115927 (2023).

28.	Yan, X. *et al.* Transoral endoscopic thyroidectomy vestibular approach for papillary thyroid microcarcinoma: an analysis of clinical outcomes. *Am J Transl Res* **14**, 7907–7915 (2022).

29.	Jiang, J. *et al.* Novel suspension system for gasless transoral vestibular thyroidectomy. *Surg Endosc* **37**, 1070–1076 (2023).

30.	Zhou, S. *et al.* Defining the Thyroid-RLN Entry Triangle for Enhanced Recurrent Laryngeal Nerve Exposure in TOETVA: A Retrospective Study. *J Otolaryngol Head Neck Surg* **53**, 19160216241301328 (2024).

31.	Deng, Z., Hu, X., Zeng, F., Guo, Y. & Peng, X. Determining the optimal port placement for transoral endoscopic thyroidectomy vestibular approach in a retrospective study. *Sci Rep* **15**, 6948 (2025).

32.	Zhang, D., Wan, L., Frattini, F. & Dionigi, G. Increased acute-phase reaction after the transoral endoscopic thyroidectomy vestibular approach (TOEVA) compared with minimally invasive video-assisted thyroidectomy (MIVAT) for unilateral thyroidectomy. *Surgery today* **55**, 969‐976 (2025).

33.	Sun, H., Chu, Y., Zhang, G., Zheng, G. & Zheng, H. The ambulatory transoral endoscopic thyroidectomy vestibular approach is safe and economical for patients with thyroid nodules. *Front Endocrinol (Lausanne)* **14**, 1116280 (2023).

34.	Wen, X. *et al.* ‘Three-in-One Wonder’: A Retrospective Cohort Study on Modified Robotic-Assisted Transoral Thyroidectomy. *J Otolaryngol Head Neck Surg* **53**, 19160216241304384 (2024).

35.	Wang, Y., Zhang, L., Huang, J. & Wang, L. Preoperative application of carbon nanoparticles in transoral endoscopic thyroidectomy vestibular approach for papillary thyroid cancer. *Front Oncol* **13**, 1120411 (2023).

36.	Zhang, W.-D. *et al.* Transoral Endoscopic Thyroidectomy Vestibular Approach Versus Gasless Transaxillary Endoscopic Thyroidectomy for Patients With Unilateral Papillary Thyroid Carcinoma: A Retrospective Study. *Surg Laparosc Endosc Percutan Tech* **33**, 347–350 (2023).

37.	Zhang, W.-D., Le, Q., Yu, K.-J., Wang, Y.-C. & Wu, X.-J. Application of Carbon Nanoparticles in Transoral Endoscopic Thyroidectomy Vestibular Approach for Unilateral Papillary Thyroid Cancer. *Surg Laparosc Endosc Percutan Tech* **34**, 301–305 (2024).

38.	Jin, X., Huang, Z., Guo, P. & Yuan, R. TOETVA: a single surgeon’s learning curve and a case report of CASTLE thyroid tumor. *Langenbecks Arch Surg* **408**, 398 (2023).

39.	Wang, M. *et al.* Transoral vestibular robotic thyroidectomy in pediatric thyroid disease: 5 case reports. *Endocrine* **88**, 523–528 (2025).

40.	Deng, Y. *et al.* Transoral endoscopic thyroid lobectomy and ipsilateral central neck lymph node dissection vestibular approach: analysis of the learning curve and clinical outcomes evaluation. *Front Endocrinol (Lausanne)* **15**, 1498797 (2024).

41.	Zhang, X. *et al.* Comparison of Transoral Endoscopic Thyroidectomy via the Vestibular Approach and Conventional Open Thyroidectomy for Papillary Thyroid Carcinoma: A Propensity Score-Matched Analysis. *Ann Surg Oncol* **32**, 6765–6773 (2025).

42.	Qian, L. *et al.* The impact of body mass index on operative time in transoral endoscopic thyroidectomy vestibular approach for thyroid cancer. *Endocrine* **84**, 179–184 (2024).

43.	Sheng, X., Fang, J., Chen, G., Li, W. & Wang, S. A suspension surgical space construction method involving a gasless transoral endoscopic thyroidectomy vestibular approach: a single-center experience. *World J Surg Oncol* **23**, 58 (2025).

44.	Wu, B., Chen, X. & Huang, J. Antibiotic Use Can be De-escalated During Transoral Endoscopic Thyroidectomy: A Bacterial Culture-Based Study. *Ear Nose Throat J* **104**, NP669–NP674 (2025).

45.	Zhou, J., Li, X., Xia, F. & Su, D. Enhanced cosmetic outcomes with central venous catheter drainage in transoral vestibular endoscopic thyroidectomy: a prospective randomized-controlled trial. *Journal of robotic surgery* **19**, 498 (2025).

46.	Zhang, Y., Hu, H., Tan, J.-T., Yao, T.-J. & Qian, L.-Y. A comparative study of transoral and submental versus vestibular endoscopic thyroidectomy in patients with papillary thyroid carcinoma. *Surg Endosc* **40**, 512–518 (2026).

47.	Xu, T. *et al.* Surgical efficiency, safety, and quality of life among four various approaches for T1 stage papillary thyroid carcinoma: a prospective cohort study. *Surg Endosc* **40**, 1984–1998 (2026).

48.	Qin, J. *et al.* Comparison of surgical trauma between three endoscopic thyroidectomy approaches and open surgery. *Front Oncol* **15**, 1677239 (2025).

49.	Park, J.-O. & Sun, D.-I. Transoral endoscopic thyroidectomy: our initial experience using a new endoscopic technique. *SURGICAL ENDOSCOPY AND OTHER INTERVENTIONAL TECHNIQUES* **31**, 5436–5443 (2017).

50.	Yi, J. W. *et al.* Transoral endoscopic surgery for papillary thyroid carcinoma: initial experiences of a single surgeon in South Korea. *Ann Surg Treat Res* **95**, 73–79 (2018).

51.	Kim, H. K. *et al.* Transoral Robotic Thyroidectomy for Papillary Thyroid Carcinoma: Perioperative Outcomes of 100 Consecutive Patients. *WORLD JOURNAL OF SURGERY* **43**, 1038–1046 (2019).

52.	Park, J.-O., Anuwong, A., Kim, M. R., Sun, D.-I. & Kim, M.-S. Transoral endoscopic thyroid surgery in a Korean population. *Surg Endosc* **33**, 2104–2113 (2019).

53.	Park, J.-O. *et al.* Gasless transoral endoscopic thyroidectomy vestibular approach (gasless TOETVA). *Surg Endosc* **33**, 3034–3039 (2019).

54.	Yi, J. W., Kim, S. -j. & Lee, K. E. Evaluation of the efficacy of postoperative antibiotic treatment in transoral endoscopic thyroidectomy: a prospective randomised controlled trial. *BRITISH JOURNAL OF ORAL & MAXILLOFACIAL SURGERY* **58**, 334–340 (2020).

55.	Han, S. *et al.* Functional Voice and Swallowing Outcome Analysis After Thyroid Lobectomy: Transoral Endoscopic Vestibular Versus Open Approach. *World J Surg* **44**, 4127–4135 (2020).

56.	Tae, K. *et al.* Sensory change in the chin and neck after transoral thyroidectomy: Prospective study of mental nerve injury. *Head Neck* **42**, 3111–3117 (2020).

57.	Hong, Y. T., Ahn, J.-H., Kim, J. H., Yi, J. W. & Hong, K. H. Bi-institutional experience of transoral endoscopic thyroidectomy: Challenges and outcomes. *Head Neck* **42**, 2115–2122 (2020).

58.	Kim, M. *et al.* Can voice pitch be preserved in patients after transoral endoscopic thyroidectomy vestibular approach? *Journal of clinical medicine* **9**, 1‐11 (2020).

59.	Ahn, J.-H., Kim, J. H., Yi, J. W. & Hur, M. H. Comparison between the 4K ultra-high definition (UHD) and high definition (HD) endoscopic systems for transoral endoscopic thyroidectomy. *Gland Surg* **9**, 229–237 (2020).

60.	Chai, Y. J., Chae, S., Oh, M. Y., Kwon, H. & Park, W. S. Transoral Endoscopic Thyroidectomy Vestibular Approach (TOETVA): Surgical Outcomes and Learning Curve. *J Clin Med* **10**, (2021).

61.	Lee, M.-C., Ahn, J., Choi, I. J., Lee, B.-C. & Ryu, J. Comparative Study of Gasless Transaxillary, Bilateral Axillo-Breast, Unilateral Axillo-Breast With Carbon Dioxide Insufflation, Retroauricular, and Transoral Vestibular Endoscopic Thyroidectomy Approaches at a Single Institution: A Retrospective Analysis and Lessons Learned. *Clin Exp Otorhinolaryngol* **15**, 283–291 (2022).

62.	Kim, S., Kim, G.-W. & Hong, Y. T. Phonetic features of patients with transoral endoscopic thyroidectomy. *Auris Nasus Larynx* **50**, 593–600 (2023).

63.	Park, J.-O. *et al.* Transoral endoscopic thyroidectomy using a self-retaining retractor as an alternative to carbon dioxide gas insufflation: A comparative analysis of 131 cases. *Oral Oncol* **121**, 105463 (2021).

64.	Lee, M. J., Oh, M. Y., Lee, J.-M., Sun, J. & Chai, Y. J. Comparative surgical outcomes of transoral endoscopic and robotic thyroidectomy for thyroid carcinoma: a propensity score-matched analysis. *Surg Endosc* **37**, 1132–1139 (2023).

65.	Lee, J. H., Choi, H. J., Woo, J.-W. & Jung, E.-J. Robotic versus endoscopic transoral thyroidectomy in papillary thyroid cancer: A comparative analysis of surgical outcomes in 240 consecutive patients. *Head Neck* **45**, 827–837 (2023).

66.	Lee, J. H. & Gwak, H. Oncologic adequacy and outcomes of open, transoral endoscopic, and robotic thyroidectomy for papillary thyroid carcinoma: a propensity score-matched analysis. *World J Surg Oncol* **24**, 5 (2025).

67.	Le, Q. V., Ngo, D. Q., Tran, T. D. & Ngo, Q. X. Transoral Endoscopic Thyroidectomy Vestibular Approach: An Initial Experience in Vietnam. *Surg Laparosc Endosc Percutan Tech* **30**, 209–213 (2020).

68.	Ngo, D. Q., Le, D. T., Hoang, G. N., Ngo, Q. X. & Le, Q. V. Case Report: Transoral Endoscopic Thyroidectomy via Vestibular Approach in Pediatric Thyroid Cancer. *Front Pediatr* **9**, 765278 (2021).

69.	Xuan Nguyen, H., Nguyen, H. X., Thi Hoang, H. & Van Le, Q. Quality of Life and Surgical Outcome of Transoral Endoscopic Thyroidectomy Vestibular Approach (TOETVA) versus Open Thyroid Surgery: Experience from a Single Center in Vietnam. *J Thyroid Res* **2022**, 2381063 (2022).

70.	Nguyen, H. X., Nguyen, H. X., Le, A. D. & Van Le, Q. Comparison of Transoral Endoscopic Thyroidectomy Vestibular Approach and Conventional Open Thyroidectomy in Benign Thyroid Tumors. *Indian J Surg Oncol* **13**, 178–183 (2022).

71.	Nguyen, H. X., Nguyen, H. X., Nguyen, T. T. P. & Van Le, Q. Transoral endoscopic thyroidectomy by vestibular approach in Viet Nam: surgical outcomes and long-term follow-up. *Surg Endosc* **36**, 4248–4254 (2022).

72.	Ngo, Q. X. *et al.* Transoral endoscopic thyroidectomy vestibular approach versus conventional open thyroidectomy for the treatment of benign thyroid tumours: A prospective cohort study. *J Minim Access Surg* **20**, 403–407 (2024).

73.	Xuan, H. N., Anh, T. D., Xuan, H. N., Thai, D. P. & Le Van, Q. Occult Central Lymph Node Metastasis in cN0 Papillary Thyroid Carcinoma Patients Undergoing TOETVA Procedure. *J Thyroid Res* **2023**, 4779409 (2023).

74.	Nguyen, K. A. & Nguyen, N. D. Transoral endoscopic thyroidectomy with or without central neck dissection. *Am J Otolaryngol* **44**, 103728 (2023).

75.	Ngo, D. Q., Le, D. T., Ngo, Q. X. & Van Le, Q. Transoral endoscopic thyroidectomy vestibular approach as a novel technique for pediatric populations: Results from a single surgeon. *Front Endocrinol (Lausanne)* **14**, 1177633 (2023).

76.	Nguyen, H. X., Nguyen, H. X., Nguyen, T. N., Luu, L. H. & Le, Q. V. Male Thyroid Cancer Patients Treated by Transoral Endoscopic Thyroidectomy Vestibular Approach (TOETVA): Surgical Outcomes and Distinct Experiences. *INDIAN JOURNAL OF SURGICAL ONCOLOGY* https://doi.org/10.1007/s13193-025-02284-x (2025) doi:10.1007/s13193-025-02284-x.

77.	Nguyen, H. X., Nguyen, H. X., Nguyen, T. N., Luu, L. H. & Van Le, Q. Male Thyroid Cancer Patients Treated by Transoral Endoscopic Thyroidectomy Vestibular Approach (TOETVA): Surgical Outcomes and Distinct Experiences. *Indian J Surg Oncol* **16**, 1738–1744 (2025).

78.	Ngo, D. Q., Truong, D. S., Le, D. T. & Hoang, T. M. Transoral endoscopic thyroidectomy vestibular approach for pT1b papillary thyroid carcinoma: surgical and oncological outcomes. *Oral Oncol* **167**, 107406 (2025).

79.	Ngo, D. Q., Le, D. T., Ngo, Q. X. & Le, Q. V. Surgical outcomes of modified versus conventional transoral endoscopic thyroidectomy vestibular approach for papillary thyroid carcinoma. *Oral Oncol* **175**, 107908 (2026).

80.	Ngo, D. Q. *et al.* Technical and therapeutic evaluation of endoscopic treatment of early papillary thyroid cancer through oral vestibular approach. *Oral Oncol* **180**, 108061 (2026).

81.	Sivakumar, T. & Amizhthu, R. A. Transoral endoscopic total thyroidectomy vestibular approach: A case series and literature review. *J Minim Access Surg* **14**, 118–123 (2018).

82.	Kumar, C. *et al.* Endoscopic thyroidectomy: which one is the better technique for the beginners? *Journal of minimal access surgery* **17**, 337‐341 (2021).

83.	Bhargav, P. R. K., Sabaretnam, M. & Amar, V. Endoscopic Thyroidectomy Through Oro-Vestibular Route (ETOVR). *INDIAN JOURNAL OF SURGERY* **82**, 598–603 (2020).

84.	Chand, G. *et al.* Natural Orifice Endoscopic Thyroidectomy via Transoral Vestibular Approach (TOVA): Single Surgeon Experience from North India. *Indian J Otolaryngol Head Neck Surg* **73**, 160–166 (2021).

85.	Bhandarwar, A. *et al.* Endoscopic Thyroidectomy: A 6-Year Experience from a Tertiary Care Teaching Hospital in Western India. *J Laparoendosc Adv Surg Tech A* **33**, 728–737 (2023).

86.	Shah, S. Transoral Endoscopic Thyroidectomy Vestibular Approach (TOETVA)-a Case Series Analysis. *INDIAN JOURNAL OF SURGERY* https://doi.org/10.1007/s12262-022-03558-2 (2022) doi:10.1007/s12262-022-03558-2.

87.	Harsha, M. P., Padha, K., Dwivedi, D. & Mateen, M. A. Natural Orifice Trans-oral, Trans-vestibular Endoscopic Thyroidectomy (NO-TOTVET): a Case Series. *Indian J Surg Oncol* **15**, 721–725 (2024).

88.	Chand, G., Godi, P., Kumari, M. & Yadav, A. Trans-Oral Endoscopic Thyroidectomy via Vestibular Approach (TOETVA): Single-Surgeon Experience of more than 100 Cases. *INDIAN JOURNAL OF SURGERY* https://doi.org/10.1007/s12262-025-04302-2 (2025) doi:10.1007/s12262-025-04302-2.

89.	Rege, S. A., Vasa, D., Shah, S. & Salvi, V. Endoscopic thyroidectomy - Is it safe and reliable? - Experience and results at a tertiary care centre in India. *J Minim Access Surg* https://doi.org/10.4103/jmas.jmas_309_25 (2026) doi:10.4103/jmas.jmas_309_25.

90.	Yadav, S. K., Johri, G., Shekhar, S., Agarwal, P. & Sharma, D. The Learning Curve for Transoral Endoscopic Thyroidectomy Without Neuromonitoring: Analysis of First 103 Cases From India. *OTO Open* **9**, e70142 (2025).

91.	Russell, J. O. *et al.* Transoral thyroidectomy and parathyroidectomy - A North American series of robotic and endoscopic transoral approaches to the central neck. *Oral Oncol* **71**, 75–80 (2017).

92.	Russell, J. O. *et al.* Anterior cervical incision-sparing thyroidectomy: Comparing retroauricular and transoral approaches. *Laryngoscope Investig Otolaryngol* **3**, 409–414 (2018).

93.	Fernandez-Ranvier, G. *et al.* Transoral Endoscopic Thyroidectomy Vestibular Approach: A Single-institution Experience of the First 50 Cases. *Surg Innov* **27**, 439–444 (2020).

94.	Fernandez Ranvier, G. *et al.* International Multi-institutional Experience with the Transoral Endoscopic Thyroidectomy Vestibular Approach. *J Laparoendosc Adv Surg Tech A* **30**, 278–283 (2020).

95.	G., F.-R. G. *et al.* Transoral Endoscopic Thyroidectomy Vestibular Approach (TOETVA): Treatment for Benign Thyroid Diseases. *Journal of Endocrine Surgery* **21**, 13–19 (2021).

96.	Razavi, C. R. *et al.* The variable direct cost and cost drivers of transoral endoscopic thyroidectomy vestibular approach. *Gland Surg* **10**, 521–528 (2021).

97.	Nagururu, N. V. *et al.* Long-term Quality of Life After Thyroidectomy: Transoral Endoscopic Thyroidectomy Vestibular Approach Versus Transcervical Approach. *Otolaryngol Head Neck Surg* **171**, 45–53 (2024).

98.	Chen, H.-K. *et al.* Application of transoral continuous intraoperative neuromonitoring in natural orifice transluminal endoscopic surgery for thyroid disease: a preliminary study. *Surg Endosc* **32**, 517–525 (2018).

99.	Wu, Y.-J. *et al.* What is the Appropriate Nodular Diameter in Thyroid Cancer for Extraction by Transoral Endoscopic Thyroidectomy Vestibular Approach Without Breaking the Specimens? A Surgicopathologic Study. *Surg Laparosc Endosc Percutan Tech* **28**, 390–393 (2018).

100.	Chen, Y.-H., Kim, H.-Y., Anuwong, A., Huang, T.-S. & Duh, Q.-Y. Transoral robotic thyroidectomy versus transoral endoscopic thyroidectomy: a propensity-score-matched analysis of surgical outcomes. *Surg Endosc* **35**, 6179–6189 (2021).

101.	Yeh, Y.-T. *et al.* Printing a Three-Dimensional Patient-Specific Safety Device for Reducing the Potential Risk of Mental Nerve Injury During Transoral Thyroidectomy. *World J Surg* **44**, 371–377 (2020).

102.	Wang, S.-H. *et al.* Application and advantages of a joystick-controlled robotic scope holder in transoral endoscopic thyroidectomy vestibular approach: a comparative study. *Updates Surg* **76**, 1425–1434 (2024).

103.	Liang, T.-J., Chen, I.-S., Chen, Y.-C. & Liu, S.-I. Feasibility of completion thyroidectomy via transoral endoscopic vestibular approach. *Updates Surg* **76**, 2565–2571 (2024).

104.	Uen, Y.-H., Wu, C.-W., Wen, K.-S., Lin, Y.-F. & Lin, K.-Y. Percutaneous continuous vagal stimulation provided superior neuromonitoring over the peroral electrode in transoral thyroidectomy. *Surg Endosc* **39**, 6249–6258 (2025).

105.	Kose, O. C., Turk, Y., Ozdemir, M., Makay, O. & Icoz, G. Patient Eligibility for Transoral Endoscopic Thyroidectomy Vestibular Approach in an Endemic Region. *Sisli Etfal Hastan Tip Bul* **55**, 304–309 (2021).

106.	Dinç, B., İlker Turan, M., Rıza Gündüz, U. & Haluk Belen, N. Transoral endoscopic thyroidectomy vestibular approach (TOETVA): Our outcomes from Turkey. *Turk J Surg* **36**, 340–346 (2020).

107.	Ünlü, M. T., Aygun, N., Serin, E. & Uludag, M. Comparison of transoral endoscopic thyroidectomy vestibular approach and open conventional thyroidectomy regardıng inflammatory responses, pain, and patient satisfaction: a prospective study. *Front Surg* **10**, 1281093 (2023).

108.	Dinc, B., Gunduz, U. R. & Belen, N. H. Transoral Endoscopic Thyroidectomy Vestibular Approach (TOETVA) in Thyroiditis. *Surg Laparosc Endosc Percutan Tech* **31**, 188–192 (2020).

109.	Unlu, M. *et al.* Did scarless thyroidectomy meet expectations? An evaluation in the aspect of cosmesis: a single-centre prospective study. *Journal of minimal access surgery* https://doi.org/10.4103/jmas.jmas_27_25 (2025) doi:10.4103/jmas.jmas_27_25.

110.	Unlu, M. T. *et al.* Did scarless thyroidectomy meet expectations? An evaluation in the aspect of cosmesis: A single-centre prospective study. *JOURNAL OF MINIMAL ACCESS SURGERY* **22**, 51–56 (2026).
